# β-to-β Singly Linked Subphthalocyanine Dimers with Effective π-Conjugation

**DOI:** 10.1021/acs.orglett.4c03407

**Published:** 2024-10-31

**Authors:** Daniel Holgado, Marta Gómez-Gómez, Jorge Labella, Tomás Torres

**Affiliations:** † Department of Organic Chemistry, Universidad Autónoma de Madrid, Campus de Cantoblanco, 28049 Madrid, Spain; ‡ Institute for Advanced Research in Chemical Sciences (IAdChem), Universidad Autónoma de Madrid, 28049 Madrid, Spain; § IMDEA-Nanociencia, Campus de Cantoblanco, 28049 Madrid, Spain

## Abstract

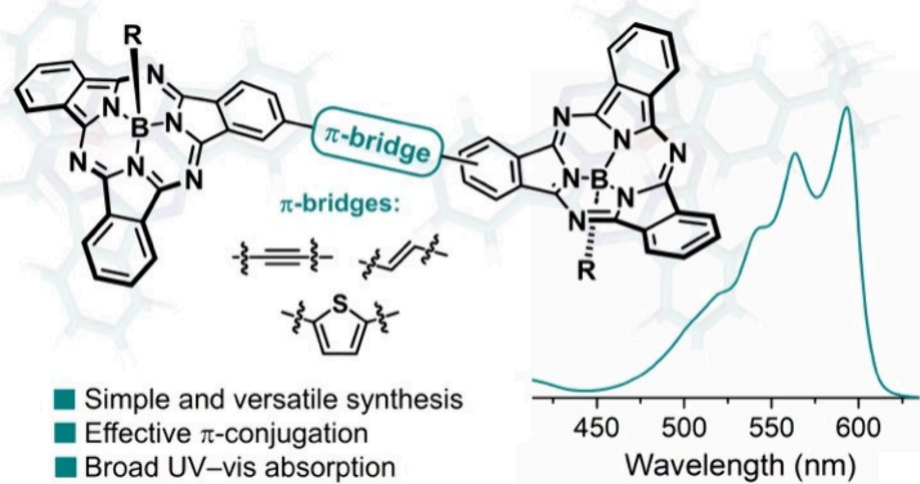

In this work, a series of π-conjugated Subphthalocyanine dimers assembled by simple π-bridges were efficiently synthesized through metal-catalyzed reactions. Despite being singly linked, these readily accessible arrays exhibit excellent π-electron communication, significantly perturbing the orbital distribution of conventional SubPcs and inducing notable alterations in their optical properties. The findings presented here demonstrate the potential of SubPcs for constructing curved porphyrin arrays with well-conjugated skeletons and intriguing functionalities.

Motivated by the efficiency of natural photosynthetic systems, π-conjugated porphyrin arrays have garnered significant attention as models for light-harvesting antenna complexes and for the study of electron excitation and charge transport.^1−3^ Owing to their highly delocalized electronic networks, they exhibit unique optical and redox properties—differing markedly from those of monomeric species—that can be exploited in cutting-edge technologies,^4^ including NIR absorption/emission,^5^ nanoelectronic devices,^6,7^ molecular magnets,^8^ and nonlinear optics.^9^ Such properties can be fine-tuned through precise control over the π-layout, including adjustments in size, symmetry, and the selection of building blocks and π-bridges.^10^ In this context, breaking the planarity of porphyrin arrays represents a new design avenue, as it imparts intriguing electronic features and opens the door to curved π-systems with novel topologies and supramolecular characteristics. Illustrative examples of this concept include the porphyrin nanorings developed by Anderson and co-workers,^11−13^ or the porphyrin arch-tapes described by Osuka and co-workers.^14,15^ Remarkably, these arrays are generally assembled from flat derivatives, predominantly porphyrins, where the curvature is induced by torsional forces. However, the use of intrinsically nonplanar porphyrinoids is a promising approach that, although seemingly intuitive, remains barely explored.

For this purpose, Subphthalocyanines (SubPcs),^16,17^ with their distinctive bowl-shaped, 14π-electron aromatic skeleton, emerge as prime candidates by virtue of their synthetic versatility and functional landscape, which extends from advanced photovoltaics and spintronics,^18,19^ to bioimaging and photodynamic therapy,^20,21^ among other applied fields.^22^ Importantly, the aromatic core—and thus the electronic distribution—of these macrocycles is susceptible to conjugative perturbation at periphery, making them ideal for creating conjugated arrays. However, despite significant progress in SubPc chemistry over the last century, very few π-conjugated SubPc arrays have been reported. In fact, beyond SubPc fused dimers,^23−26^ which are challenging to prepare and purify, the SubPc arrays reported so far are limited to dimers or trimers, β-to-β linked through large acetylenic scaffolds.^27−30^ These π-bridges, although elegant, preclude maximum π-delocalization, which is a prerequisite for achieving electronic modulation and new functions.

Herein, we synthesized a series of conjugated SubPc dimers, namely **1**–**4** (Scheme 1), singly linked by easy-to-install π-linkers that allow for strong electronic communication, inducing significant changes in the electronic distribution of the SubPc units.

**Scheme 1:**
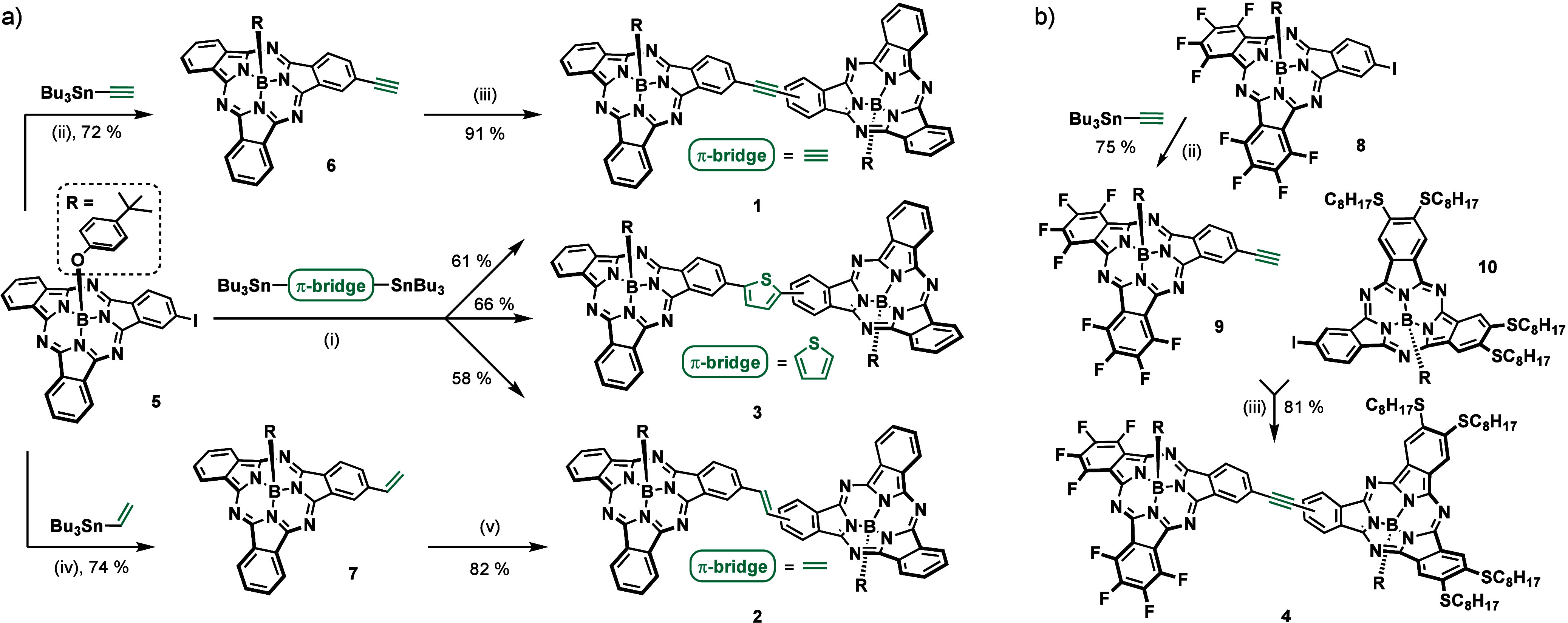
Synthetic Strategy towards (a) Homodimers **1**–**3**, and (b) Push–Pull Heterodimer **4** Reagents and conditions: (i) Pd(PPh_3_)_4_ (15 mol %), toluene, 24 h, 100 °C; (ii) Pd(PPh_3_)_4_ (15 mol %), toluene, 15 h, 55 °C; (iii) PdCl_2_(PPh_3_)_2_ (6.5 mol %), CuI (6.5 mol %), toluene/NEt_3_ (10:1), 14 h, rt; (iv) Pd(PPh_3_)_4_ (15 mol %), toluene, 15 h, 70 °C; (v) Grubbs 3rd generation catalyst (5 mol %), toluene, 24 h, 45 °C.

Since the early 1990s, ethynyl and ethenyl moieties have been pivotal π-spacers for building conjugated porphyrin arrays.^10^ Similarly, thienyl units, although not as efficient in overlapping as the previous ones, provide added-value charge-transfer processes and intermolecular interactions.^31,32^ On this basis, symmetric dimers **1**–**3** were targeted and synthesized, as shown in Scheme 1a. **1** and **2** were obtained through two routes, either one-step or stepwise, whereas dimer **3** was prepared only by the former. Both routes start from key precursor **5,** which is a monoiodinated SubPc prepared by statistical cyclotrimerization (see Supporting Information (SI)). The one-step approach involved a double Stille cross-coupling reaction between iodinated SubPc **5** and either 1,2-bis(tributylstannyl)ethyne, (*E*)-1,2-bis(tributylstannyl)ethene, or 2,5-bis(tributylstannyl)thiophene, affording dimers **1**-**3** in excellent yields (58–66%). On the other hand, the stepwise assembly of **1** and **2** was carried out by reacting **5** with tributyl(ethynyl)stannane and tributyl(vinyl)stannane, respectively, in the presence of Pd(PPh_3_)_4_ to furnish the corresponding ethynyl and ethenyl SubPcs, **6** and **7**, in 72% and 74% yields. **6** and **7** were then subjected to Sonogashira cross-coupling and olefin metathesis, yielding **1** and **2** in 91% and 82% yield, respectively. Notably, during the synthesis of **7**, small amounts of **2** were unexpectedly detected, presumably resulting from a cascade base free Heck coupling between **7** and **5**. As detailed in the SI, **1**–**3** were unambiguously characterized by NMR and mass spectroscopy. It is important to note that since key precursor **5** is intrinsically chiral and used as a racemic mixture, **1**–**3** are obtained as a mixture of two diastereoisomers. One diastereoisomer exists as a pair of enantiomers (*MM* and *PP*), while the other involves the *meso* form (*MP* or *PM*). These isomers could not be isolated from each other in a preparative manner due to their similarity. Nevertheless, the diastereoisomeric composition is clearly detected by HPLC analysis, revealing three signals with an intensity ratio of 1:2:1. As expected, this indicates that there is no diastereoselectivity in our synthesis.

To assess the π-delocalization of **1**–**3**, we studied their optical properties using UV–vis absorption and fluorescence spectroscopy in THF solution (Figure 1). Crucially, **1**–**3** exhibit a substantially different absorption profile compared to that of the nonsubstituted SubPc monomer (**H**_**12**_**SubPc-R**). As shown in Figure 1, **1–3** exhibit red-shifted, split Q-bands with two maxima in the range of 450–625 nm, as well as lower intensity Soret transitions at 300–450 nm. This contrasts sharply with the absorption of **H**_**12**_**SubPc-R**, which features a well-defined Soret band peaking at 303 nm and a typical single-peak Q-band centered at 560 nm. Hence, electronic distribution of the SubPc has been perturbed upon dimerization. This effect is also observed, although much less pronounced, in directly β-to-β linked SubPc dimers.^33^ Interestingly, the Q-band experienced a more significant red-shift in **2** (λ_max_ = 601 nm), followed by **3** (λ_max_ = 597 nm) and **1** (λ_max_ = 593 nm), suggesting that the ethenyl moiety offers slightly enhanced conjugation compared to the other bridges. Importantly, the Q-bands of **1**–**3** exhibit a notably high extinction coefficient of ca. 130 000 M^–1^ cm^–1^, more than double that of **H**_**12**_**SubPc-R** (57 000 M^–1^ cm^–1^). This absorption enhancement is attributed to the presence of two SubPc units, as well as to the increased cross-section–parallel to the transition dipole moment of SubPcs–through the π-bridge. These results clearly indicate excellent π-conjugation in **1**–**3**, underscoring the efficiency of the selected π-bridges for the peripheral π-delocalization of SubPcs. Regarding the emission properties, **1**–**3** showed a fluorescence quantum yield of 45–54% in THF, comparable to that of SubPc (45%).

**Figure 1:**
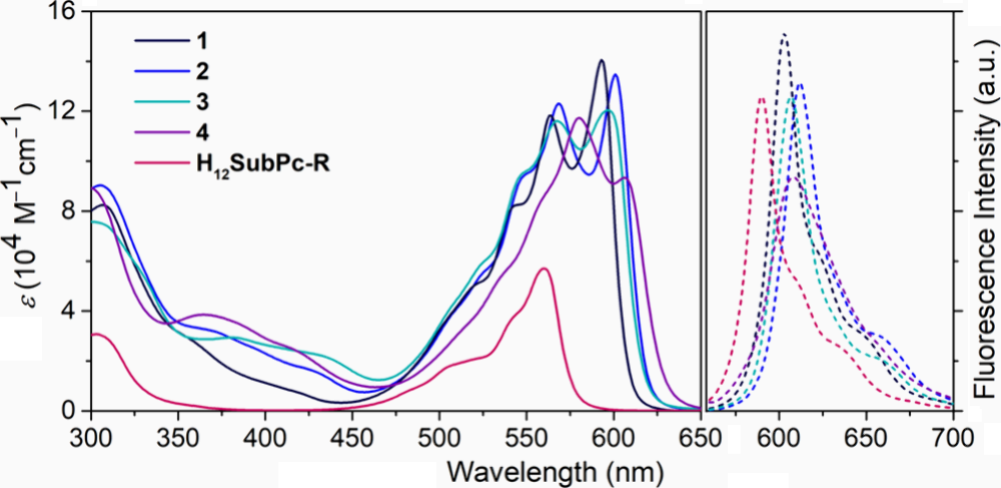
Absorption (solid lines) and emission (dashed lines) spectra of **1**–**4** and **H**_**12**_**SubPc-R** in THF. [SubPc] = 2.5 × 10^–5^ M for absorption; 1.5 × 10^–6^ M for emission (λ_exc_ = 530 nm).

Based on these results, we turned our attention to the construction of a donor–acceptor (D-A) SubPc heterodimer (**4**), aiming to create a push–pull π-conjugated architecture and induce further electronic coupling.^34,35^ To this end, ethynyl was chosen as the linker. As shown in Scheme 1b, **4** was assembled in a two-step procedure, starting with a Sonogashira cross-coupling between the electron-deficient SubPc **9**—prepared from precursor **8** through a Stille cross-coupling with tributyl(ethynyl)stannane—and the electron-rich SubPc **10**. The push–pull dimer **4** was obtained in a yield of 61% over two steps. As with previous dimers, **4** was obtained as a mixture of diastereoisomers. However, as detailed in the SI, in this case, the two isomers consist of a racemic mixture of enantiomers due to the asymmetry of the molecule. Concerning the optical properties, **4** displays a broad Q-band spanning from 450 to 640 nm, whose shape noticeably differs from that of **1**–**3** (Figure 1). This band is red-shifted compared to those of **1**–**3**, as is typical in push–pull porphyrin arrays. Another notable difference is the fluorescence quenching observed in **4** (34%) in comparison with **1**–**3**. This quenching may be attributed to photoinduced electron transfer between the two SubPc units, given their complementary electronic nature. This is in agreement with the electrostatic potential maps calculated by density functional theory (DFT), which reveal higher electronic density in the fluorinated SubPc unit (see SI).

To shed light into the origin of the spectral features of **1**–**4**, we analyzed their orbital distribution by DFT. Additionally, time-dependent density functional theory (TD-DFT) calculations were performed to assign the nature of the transitions. Figure 2a shows the selected molecular orbitals of **1**, with **2** and **3** exhibiting the same pattern (see SI), and their energy levels calculated at the B3LYP/6-31G(d,p) level of theory. Notably, there is significant participation of the π-bridge in both the HOMO, LUMO and LUMO+3. In contrast, the HOMO–1, which is close in energy to the HOMO, as well as LUMO+1 and LUMO+2 (the latter two being degenerate and close in energy to LUMO+3), are symmetrically distributed over the SubPc units. This specific orbital arrangement contrasts with that of typical SubPcs, such as **H**_**12**_**SubPc-R**, which feature a much more stabilized HOMO–1 compared to the HOMO, and degenerate LUMO and LUMO+1 levels.^22^ This is consistent with the split Q-band observed experimentally for **1**–**3**. Quantitative support for this statement came from TD-DFT, which reveals that the Q-band of **1**–**3** arises from transitions between six orbitals: HOMO, HOMO–1, LUMO, LUMO+1, LUMO+2, and LUMO+3. In contrast, the Q-band of the archetypal **H**_**12**_**SubPc-R** arises solely from HOMO → LUMO and HOMO → LUMO+1 transitions.^22^ Importantly, similar behavior is observed in **4**, although the asymmetry introduced by the push–pull structure results in a distinct orbital order, accounting for the different shape of the Q-band compared to homodimers. In the case of **4**, no orbitals with symmetric contribution by both SubPcs are observed; instead, they are localized either in the electron-deficient or electron-rich subunit. Consequently, the most stable LUMOs and HOMOs are located on the fluorinated moiety, while the higher-lying HOMOs and LUMOs are found on the thioether-equipped unit.

**Figure 2:**
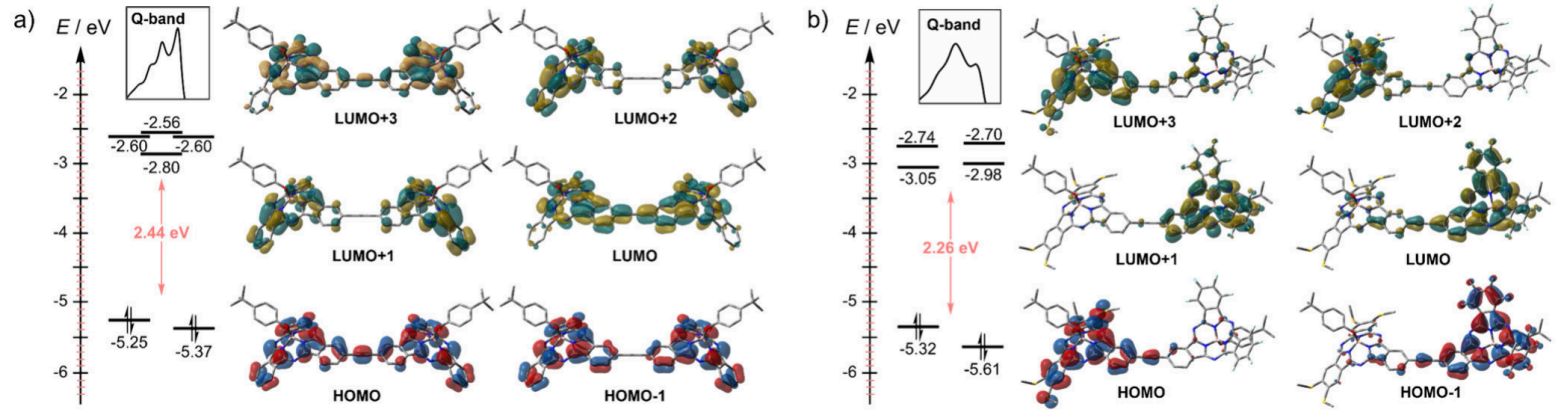
Selected molecular orbitals and their energy levels calculated by DFT at the B3LYP/6-31G(d,p) level of theory for (a) **1** and (b) **4**. Inset: Q-band shape of the corresponding dimer. Isosurface value = 0.02. Hydrogen atoms are omitted for clarity.

In summary, four SubPc dimeric arrays, including three symmetric dimers and one with a push–pull nature, have been efficiently synthesized using ethynyl, ethenyl, or thienyl as π-bridges. These compounds were assembled through straightforward metal-catalyzed reactions. Notably, despite the single-linking mode, the SubPc units exhibit strong π-conjugation, resulting in a significant perturbation of the macrocycle’s electronic structure. The conjugated SubPc dimers display broad absorptions in the visible range, with extinction coefficients reaching up to 140 000 M^–1^ cm^–1^. These optical properties can be attributed to transitions involving six key orbitals: HOMO–1, HOMO, HOMO–1, LUMO, LUMO+1, LUMO+2, and LUMO+3. Overall, this work highlights the potential of β-to-β singly linked SubPcs for advanced materials, paving the way for the exploration of complex nonplanar π-conjugated systems with tailored properties.

## Data Availability

The data underlying this study are available in the published article and its Supporting Information.

## Supporting Information Available

Experimental procedures, compound characterization data, NMR and Mass spectra, and computational details are included in the Supporting Information. (PDF)
DFT Cartesian coordinates. (PDF)
