# Supplementary material for: β-to-β Singly Linked Subphthalocyanine Dimers with Effective π-Conjugation
Source: Org Lett. 2024 Oct 31;26(44):9471–5. doi: 10.1021/acs.orglett.4c03407 (PMC11555671; doi:10.1021/acs.orglett.4c03407)
Supplement: Supplementary file 1 — ol4c03407_si_001.pdf [file ol4c03407_si_001.pdf]

Supplementary Information

β-to-β Singly-Linked Subphthalocyanine Dimers with Effective π-Conjugation

Daniel Holgado, ‡ [a] Marta Gómez-Gómez,‡[a] Jorge Labella,*[a] and Tomás Torres*[a, b, c]

[a] Department of Organic Chemistry, Universidad Autónoma de Madrid, Campus de Cantoblanco, 28049 Madrid, Spain.
[b] Institute for Advanced Research in Chemical Sciences (IAdChem), Universidad Autónoma de Madrid, 28049 Madrid, Spain.
[c] IMDEA-Nanociencia, Campus de Cantoblanco, 28049 Madrid, Spain.

e-mail: jorge.labella@uam.es, tomas.torres@uam.es

‡These authors contributed equally.

Table of content

1. Instrumentation and materials....2
2. Synthetic Procedures and Compound Data 3
   Synthesis and characterization of peripherally monoiodinated SubPcs 3
   Synthesis and characterization of asymmetric SubPcs with alkene/alkyne groups at the periphery 4
   Synthesis and characterization SubPc dimers....5
3. NMR Spectra 8
4. Mass Spectra....15
5. Computational Studies....19
   TD-DFT calculations....19
   Calculated UV-vis espectra 22
   Selected molecular orbitals and their energy levels 22
   Electrostatic potential (ESP) map....23
6. Analytical HPLC Resolution of Racemic SubPc Dimers **1**, **2**, **3** and **4** 23
7. Supporting References....25

Abbreviations

DCM: Dichloromethane; DFT: Density Functional Theory; HOMO: Highest Occupied Molecular Orbital; HPLC: High-Performance Liquid Chromatography; HR-MS: High Resolution Mass Spectrometry; LUMO: Lowest Unoccupied Molecular Orbital; MALDI-TOF: Matrix-Assisted Laser Desorption/Ionization-Time of Flight; Mp: Melting point; NMR = Nuclear Magnetic Resonance; ppm = part per million; PCM: Polarizable Continuum Model; SubPc = Subphthalocyanine; TD-DFT: Time-Dependent Density Functional Theory; THF = Tetrahydrofuran; TLC = Thin Layer Chromatography; UV-vis = Ultraviolet-visible.

1. Instrumentation and materials

The monitoring of the reactions has been carried out by thin layer chromatography (TLC), employing aluminum sheets coated with silica gel type 60 F254 (0.2 mm thick, Merck). The analysis of the TLCs was carried out with an UV lamp of 254 and 365 nm. Purification and separation of the synthesized products were performed by normal-phase column chromatography, using silica-gel 60 (230–400 mesh, 0.040–0.063 mm, Merck) as the stationary phase. Eluents along with the relative ratio in the case of solvent mixtures are indicated for each particular case. Size exclusion chromatography was performed using Bio-Beads S-X1 (styrene divinylbenzene beads, 40-80 μm bead size, Bio-Rad) with chloroform as the mobile phase.

Nuclear magnetic resonance spectra (^{1}H-, ^{13}C-, ^{11}B-, ^{19}F-NMR) were recorded on a Bruker AV-300 or Bruker DRX-500 spectrometers. Deuterated solvent employed in each case is indicated in brackets, and its residual peak was used to calibrate the spectra using literature reference δ ppm values.[1] All the experiments were recorded at room temperature. Multiplicity was indicated using the following abbreviations: s (singlet), bs (broad singlet), d (doublet), dd (doublet of doublets), t (triplet), q (quartet), m (multiplet).

High-resolution mass spectra (HR-MS) were recorded in the Interdepartmental Investigation Service of UAM, employing matrix-assisted laser desorption/ionization time-of-flight (MALDI-TOF) using a Bruker-Ultraflex-III spectrometer with a Nd:YAG laser operating at 355 nm or ultrafleXtreme spectrometer. The matrixes and internal references employed are indicated for each spectrum. Mass spectrometry data are expressed in m/z units.

Ultraviolet and visible (UV-vis) spectra were recorded using solvents in the spectroscopic grade in the Organic Chemistry Department of UAM employing a JASCO-V660 spectrophotometer. Likewise, fluorescence measurements were carried out with a JASCO-V8600 spectrofluorometer. Fluorescence quantum yields (ϕ_F) of SubPcs were determined in toluene and calculated by using the following equation:[2]

$$\phi_F^S = \phi_F^R \left(\frac{Grad_S}{Grad_R}\right)\left(\frac{\eta_S}{\eta_R}\right)^2$$

Scripts R and S indicate reference and sample, respectively. Grad is the gradient from the plot of the integrated fluorescence intensity (at exc. λ= 530 nm) versus the absorption (at the same wavelength), and η is the refractive index of the solvent. Chloro-dodecafluoroSubPc (F_{12}SubPc-Cl) in benzonitrile ($\phi_F = 0.58$) was used as reference.[3]

All reactions dealing with air or moisture sensitive compounds were carried out by standard Schlenk techniques in a dry reaction vessel under argon. Chemicals were purchased from commercial suppliers and used without further purification. Dry solvents were purchased

from commercial suppliers in anhydrous grade or thoroughly dried before use employing standard methods. Solid, hygroscopic reagents were dried in a vacuum oven before use.

The resolution of **1**, **2**, **3** and **4** were carried out by High Performance Liquid Chromatography (HPLC) using an Agilent 1200 equipment with a semi-preparative Daicel Chiralpak IC column (10 mm ø x 20 mm). The separation conditions are indicated in each case.

The synthesis and characterization of **4-iodophthalonitrile**,[4] **4,5-bis(octylthio)phthalonitrile**,[4] **5**,[6] and **10**[7] have been previously reported.

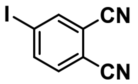

4,5-iodophthalonitrile

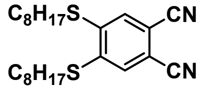

4,5-bis(octylthio)phthalonitrile

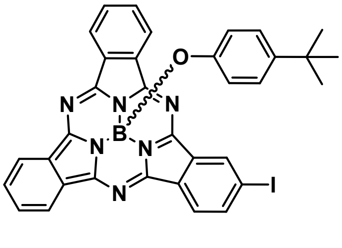

5

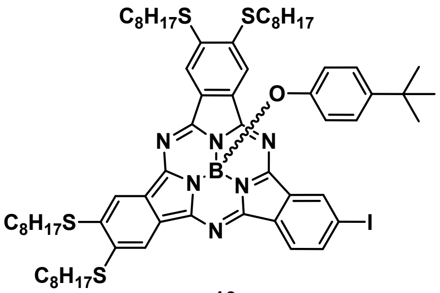

10

2. Synthetic Procedures and Compound Data

<u>Synthesis and characterization of peripherally monoiodinated SubPcs</u>

SubPc **8**:

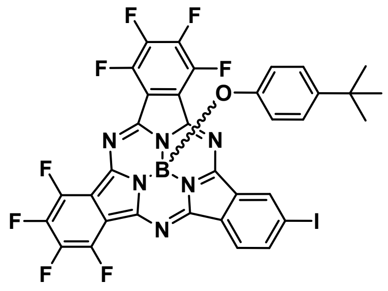

In a 50 mL two-necked round-bottomed flask, equipped with a condenser, magnetic stirrer and rubber seal, a 1.0 M solution of BCl_3 in *p*-xylene (9 mL) was added over a mixture of 4-iodophthalonitrile (0.76 g, 3 mmol) and tetrafluorophthalonitrile (1.2 g, 6 mmol) under argon atmosphere. The reaction mixture was stirred at 140 ºC for 45 min in an oil bath. The purple solution was allowed to cool to room temperature. Then, it was flushed with argon to remove the excess of BCl_3. After removal of the solvent, 4-*tert*-butylphenol (3.2 g, 21 mmol), DIPEA (*N*,*N*-diisopropylethylamine; 0.26 mL, 1.5 mmol) and dry toluene (9 mL) were added over the crude under argon atmosphere. The new reaction mixture was stirred in an oil bath at 110 °C for 24 h. The dark purple reaction slurry was dissolved in toluene and passed through a short silica plug. The solvent was removed by vacuum distillation and the resulting dark solid was purified by column chromatography on silica gel using toluene/heptane 3:2 as eluent. The product is obtained as a bright purple solid. Yield: 12 % (290 mg).

^{1}H NMR ($CDCl_3$, 300 MHz): δ 9.23 (d, 1H $^{4}J_{H\text{-}H}$ = 1.5 Hz), 8.59 (d, 1H, $^{3}J_{H\text{-}H}$ = 8.4 Hz), 8.29 (dd, 1H, $^{3}J_{H\text{-}H}$ = 8.4 Hz, $^{4}J_{H\text{-}H}$ = 1.5 Hz), 6.79 (d, 2H, $^{3}J_{H\text{-}H}$ = 8.7 Hz), 5.27 (d, 2H, $^{3}J_{H\text{-}H}$ = 8.7 Hz), 1.10 (s, 9H); **^{13}C NMR** ($CDCl_3$, 75 MHz): δ 153.4, 152.2, 149.2, 144.9, 140.1, 132.6, 132.1, 130.3, 126.2, 124.2, 117.8, 97.8, 34.1, 31.4; **^{11}B NMR** ($CDCl_3$, 160 MHz) δ -15.0; **^{19}F NMR** ($CDCl_3$, 282 MHz): δ -137.9 (m, 4F), -148.9 (m, 4F); **UV-vis** (THF): λ (nm) (log ε/dm^3 mol^{-1} cm^{-1}) = 570 (4.8), 512 (sh), 303 (4.5); **HRMS** (MALDI-TOF) m/z: [M]+ Calcd for $C_{34}H_{16}BF_8IN_6O$ 814.0396; Found 814.0414; **Mp** > 250 ºC.

Synthesis and characterization of asymmetric SubPcs with alkene/alkyne groups at periphery

General procedure for coupling vinyl/ethynyl groups on the SubPc periphery

In a 25 mL Schlenk tube, the corresponding asymmetric monoiodinated SubPc (**5** for preparing both **6** and **7**, and **8** for the synthesis of **9**; 0.030 mmol, 1 equiv), $Pd(PPh_3)_4$ (0.0045 mmol, 0.15 equiv), the corresponding stannane (tributyl(vinyl)stannane in the case of **7**, and tributyl(ethynyl)stannane for synthesizing **6** and **9**; 0.036 mmol, 1.2 equiv) and dry toluene (0.015 M) were added under argon. The reaction mixture was stirred in an oil bath for 15 h (at 70 ºC in the case of using tributyl(vinyl)stannane and 55 ºC for tributyl(ethynyl)stannane). The reaction slurry was diluted with DCM (20 mL), washed with water (2 x 20 mL) and dried over $MgSO_4$. The solvent was removed by vacuum distillation and the resulting dark solid was subjected to column chromatography (using the eluent mentioned in each case). Total purification was achieved by size exclusion chromatography.

SubPc **6**:

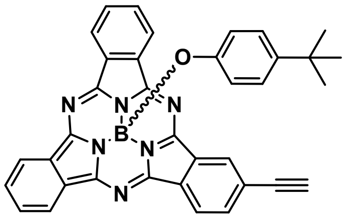

Column chromatography eluent: DCM. The product was precipitated in a DCM/MeOH mixture, obtaining the compound as a purple powder. Yield: 72 % (12 mg).

^{1}H NMR ($CDCl_3$, 300 MHz): δ 8.99 (bs, 1H), 8.88-8.80 (m, 4H), 8.78 (d, 1H, $^{3}J_{H\text{-}H}$ = 8.1 Hz), 7.96 (dd, 1H, $^{3}J_{H\text{-}H}$ = 8.1 Hz, $^{4}J_{H\text{-}H}$ = 1.2 Hz), 7.93-7.87 (m, 4H), 6.76 (d, 2H, $^{3}J_{H\text{-}H}$ = 9.0 Hz), 5.30 (d, 2H, $^{3}J_{H\text{-}H}$ = 9.0 Hz), 3.32 (s, 1H), 1.08 (s, 9H); **^{13}C NMR** ($CDCl_3$, 75 MHz) δ 152.4, 151.8, 150.5, 150.1, 143.8, 133.0, 131.3, 130.8, 130.1, 126.3, 125.9, 123.5, 122.4, 122.2, 117.9, 83.7, 79.7, 34.0, 31.5; **^{11}B NMR** ($CDCl_3$, 160 MHz) δ -14.8; **UV-vis** (THF): λ (nm) (log ε/dm^3 mol^{-1} cm^{-1}) = 569 (4.8), 528 (sh), 307 (4.5); **HRMS** (MALDI-TOF) m/z: [M]+ Calcd for $C_{36}H_{25}BN_6O$ 568.2184; Found 568.2204; **Mp** > 250 ºC.

SubPc **7**:

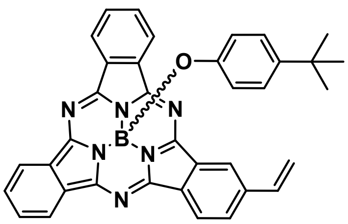

Column chromatography eluent: DCM. The product was precipitated in a DCM/MeOH mixture, obtaining the compound as a purple powder. Yield: 74 % (13 mg). Heck coupling is also observed (yield: 5%, 2 mg).

^{1}H NMR ($CDCl_3$, 300 MHz): δ 8.86-8.81 (m, 5H), 8.77 (d, 1H, $^3J_{H-H}$ = 8.1 Hz), 7.95 (dd, 1H, $^3J_{H-H}$ = 8.1 Hz, $^4J_{H-H}$ = 1.5 Hz), 7.90-7.85 (m, 4H), 7.04 (dd, 1H, $^3J_{H-H}$ = 17.7 Hz, $^3J_{H-H}$ = 11.1 Hz), 6.76 (d, 2H, $^3J_{H-H}$ = 8.7 Hz), 6.09 (d, 1H, $^3J_{H-H}$ = 17.7 Hz,), 5.49 (d, 1H, $^3J_{H-H}$ = 11.1 Hz), 5.32 (d, 2H, $^3J_{H-H}$ = 8.7 Hz), 1.08 (s, 9H); **^{13}C NMR** ($CDCl_3$, 75 MHz): δ 151.7, 151.6, 151.5, 151.5, 151.4, 150.2, 143.7, 139.6, 136.7, 131.7, 131.2, 130.2, 129.9, 127.9, 125.8, 122.4, 120.0, 117.9, 116.4, 34.0, 31.5; **^{11}B NMR** ($CDCl_3$, 160 MHz,) δ -14.8; **UV-vis** (THF): λ (nm) (log ε/dm^3 mol^{-1} cm^{-1}) = 567 (4.7), 512 (sh), 300 (4.4); **HRMS** (MALDI-TOF) m/z: [M]+ Calcd for $C_{36}H_{27}BN_6O$ 570.2340; Found 570.2358; **Mp** > 250 ºC.

SubPc **9**:

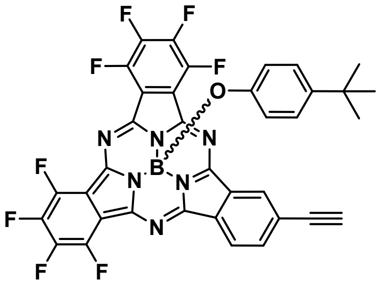

Column chromatography eluent: toluene/heptane 8:1. In this case, the product was obtained as a purple solid that can be manipulated without need of precipitation. Yield: 75 % (16 mg).

^{1}H NMR ($CDCl_3$, 300 MHz): δ 8.99 (bs, 1H), 8.81 (dd, 1H, $^3J_{H-H}$ = 8.1 Hz, $^5J_{H-H}$ = 0.9 Hz), 8.06 (dd, 1H, $^3J_{H-H}$ = 8.1 Hz, $^4J_{H-H}$ = 1.2 Hz), 6.79 (d, 2H, $^3J_{H-H}$ = 9.0 Hz), 5.28 (d, 2H, $^3J_{H-H}$ = 9.0 Hz), 3.40 (s, 1H), 1.10 (s, 9H); **^{13}C NMR** ($CDCl_3$, 75 MHz) δ 153.3, 149.2, 144.8, 134.7, 131.3, 130.7, 126.9, 126.2, 125.5, 123.0, 117.8, 83.0, 81.1, 34.1, 31.4; **^{11}B NMR** ($CDCl_3$, 160 MHz) δ -15.0; **^{19}F NMR** ($CDCl_3$, 282 MHz): δ -138.0 (m, 4F), -149.0 (m, 4F); **UV-vis** (THF): λ (nm) (log ε/dm^3 mol^{-1} cm^{-1}) = 572 (4.8), 527 (sh), 303 (4.5); **HRMS** (MALDI-TOF) m/z: [M]+ Calcd for $C_{36}H_{17}BF_8N_6O$ 712.1430; Found 712.1459; **Mp** > 250 ºC.

Synthesis and characterization SubPc dimers

Method A: Formation of SubPc dimers via double Stille coupling

A 25 mL Schlenk tube was charged with **5** (0.030 mmol, 1 equiv), $Pd(PPh_3)_4$ (0.0045 mmol, 0.15 equiv), and the corresponding stannane (bis(tributylstannyl)acetylene for preparing **1**; *trans*-1,2-bis(tributylstannyl)ethene in the case of **1** and 2,5-bis(tributylstannyl)thiophene for **3**; 0.036 mmol, 1.2 equiv) under argon atmosphere. Then, dry and deoxygenated toluene (0.015 M) was added, and the reaction mixture was stirred at 100 ºC for 24 h in an oil bath. Once the reaction is completed, the crude was cooled to room temperature, diluted in DCM (20 mL), washed with water (2 × 20 mL), and dried over anhydrous magnesium sulfate. After removing the solvent by vacuum distillation, a dark solid is obtained. That solid was subjected to column chromatography, using the eluent mentioned in each case. Finally, purification was achieved by size exclusion chromatography. The resulting solid was precipitated in a DCM/MeOH mixture, obtaining the compound as a purple powder.

Method B: Formation of SubPc dimers via metathesis reaction

A 10 mL Schlenk tube was charged with **7** (8.56 mg, 0.015 mmol) and 3rd generation Grubbs catalyst (0.66 mg, 0.8 μmol) under argon atmosphere. Then, dry and deoxygenated toluene (1 mL) was added, and the reaction mixture was stirred in an oil bath at 45 ºC for 24 h. The solvent was removed by vacuum distillation and the resulting dark solid was subjected to column chromatography, using the eluent mentioned in each case. Finally, the compound was further by size exclusion chromatography. The resulting solid is precipitated in a DCM/MeOH mixture, obtaining the compound as a purple powder.

Method C: Formation of SubPc dimers via Sonogashira reaction

In a 25 mL Schlenk flask, equipped with a magnetic stirrer, the corresponding alkyne-SubPc (**6** in the case of **1**, and **9** for obtaining **4**; 0.020 mmol, 1 equiv), the corresponding monoiodated SubPc (**5** or **10**, respectively; 0.020 mmol, 1 equiv), $PdCl_2(PPh_3)_2$ (0.0013 mmol, 0.065 equiv), and CuI (0.0013 mmol, 0.065 equiv) were placed under argon atmosphere. Then, a dry and deoxygenated mixture of toluene/NEt_3 10:1 (0.013 M) was added, and the resulting mixture was stirred at room temperature for 14 h. After that, the crude was dissolved in DCM and passed through a short celite plug. The solvent was removed by vacuum distillation and the resulting dark solid was subjected to column chromatography, using the eluent mentioned in each case. Finally, the eluted fraction was further purified by size exclusion chromatography. The resulting solid was recrystallized from a DCM/MeOH mixture, affording the desired product as a purple powder.

SubPc **1**:

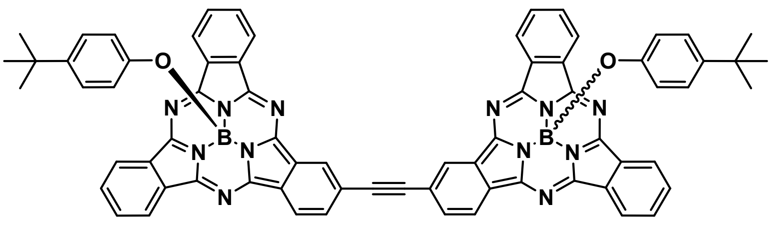

Column chromatography eluent: toluene/ethyl acetate 20:1. Yield: 61 % (10 mg; method A), 91 % (20 mg; method C).

^{1}H NMR ($CDCl_3$, 300 MHz): δ 9.12 (bs, 2H), 8.88-8.84 (m, 10H), 8.09 (dd, 2H, $^3J_{H-H}$ = 8.1 Hz, ^4JH-H = 1.2 Hz), 7.95-7.89 (m, 8H), 6.78 (d, 4H, $^3J_{H-H}$ = 8.7 Hz), 5.33 (d, 4H, $^3J_{H-H}$ = 8.7 Hz), 1.09 (s, 18H); **^{13}C NMR** ($CDCl_3$, 75 MHz) δ 152.4, 151.9, 150.7, 150.2, 143.8, 132.6, 130.1, 125.9, 124.4, 122.5, 117.9, 34.0, 31.5; **^{11}B NMR** ($CDCl_3$, 160 MHz) δ -14.8; **UV-vis** (THF): λ (nm) (log ε/dm^3 mol^{-1} cm^{-1}) = 593 (5.1), 564 (5.1), 542 (sh), 307 (4.9); **HRMS** (MALDI-TOF) m/z: [M]+ Calcd for $C_{70}H_{48}B_2N_{12}O_2$ 1110.4224; Found 1110.4219; **Mp** > 250 ºC.

SubPc **2**:

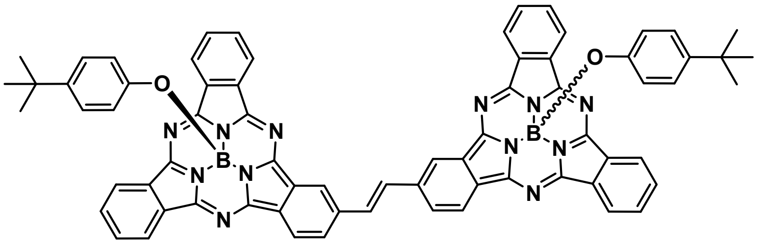

Column chromatography eluent: toluene/ethyl acetate 20:1. Yield: 58 % (10 mg; method A), 82 % (7 mg; method B).

^{1}H NMR ($CDCl_3$, 300 MHz): δ 9.05 (s, 1H), 9.03 (s, 1H), 8.88-8.81 (m, 10H), 8.10 (dd, 2H, $^{3}J_{H-H}$ = 8.4 Hz, $^{4}J_{H-H}$ = 1.5 Hz), 7.91-7.84 (m, 8H), 7.74 (s, 1H), 7.74 (s, 1H), 6.80 (d, 4H, $^{3}J_{H-H}$ = 8.7 Hz), 5.38 (d, 4H, $^{3}J_{H-H}$ = 8.7 Hz), 1.09 (s, 18H); **^{13}C NMR** ($CDCl_3$, 75 MHz) δ 151.9, 150.3, 143.8, 139.0, 131.3, 130.7, 130.0, 128.3, 125.9, 122.7, 122.4, 120.5, 117.9, 34.0, 31.5; **^{11}B NMR** ($CDCl_3$, 160 MHz) δ -14.7; **UV-vis** (THF): λ (nm) (log ε/dm^3 mol^{-1} cm^{-1}) = 601 (5.1), 569 (5.1), 546 (sh), 306 (5.0); **HRMS** (MALDI-TOF) m/z: [M]+ Calcd for $C_{70}H_{50}B_2N_{12}O_2$ 1112.4381; Found 1112.4396; **Mp** > 250 ºC.

SubPc **3**:

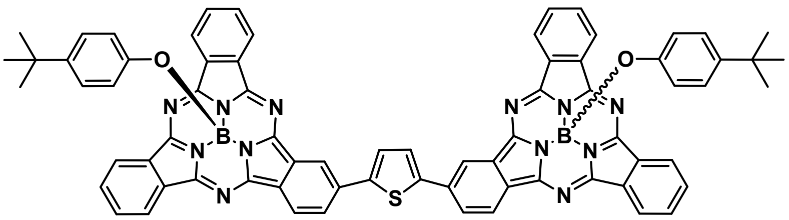

Column chromatography eluent: toluene/ethyl acetate 20:1. Yield: 66 % (12 mg; method A).

^{1}H NMR ($CDCl_3$, 300 MHz): δ 9.13 (bs, 2H), 8.88-8.81 (m, 10H), 8.18 (dt, 2H, $^{3}J_{H-H}$ = 8.4 Hz, $^{4}J_{H-H}$ = 1.5 Hz), 7.92-7.85 (m, 8H), 7.67 (s, 1H), 7.67 (s, 1H), 6.80 (d, 4H, $^{3}J_{H-H}$ = 9.0 Hz), 5.38 (d, 4H, $^{3}J_{H-H}$ = 9.0 Hz), 1.09 (s, 18H); **^{13}C NMR** ($CDCl_3$, 125 MHz): δ 152.1, 151.9, 151.5, 151.2, 150.2, 144.5, 143.8, 135.8, 131.9, 131.2, 131.1, 130.1, 129.6, 129.2, 128.4, 127.2, 126.1, 125.9, 125.4, 122.9, 122.4, 118.9, 117.9, 34.0, 31.5; **^{11}B NMR** ($CDCl_3$, 96 MHz) δ -14.8; **UV-vis** (THF): λ (nm) (log ε/dm^3 mol^{-1} cm^{-1}) = 598 (5.1), 567 (5.1), 544 (sh), 302 (4.9); **HRMS** (MALDI-TOF) m/z: [M]+ Calcd for $C_{72}H_{50}B_2N_{12}O_2S$ 1168.4102; Found 1168.4112; **Mp** > 250 ºC.

SubPc **4**:

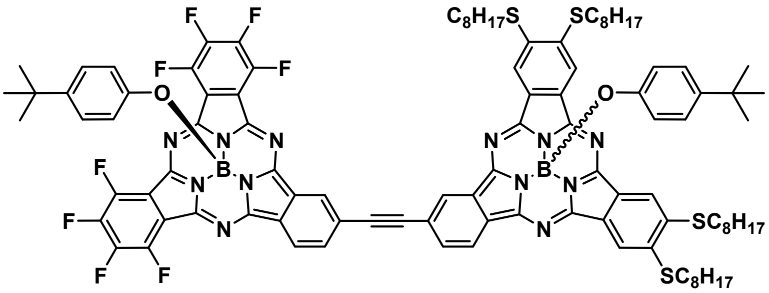

Column chromatography eluent: toluene/heptane 10:1. Yield: 81 % (30 mg; method C).

^{1}H NMR ($CDCl_3$, 300 MHz): δ 9.12-9.07 (m, 2H), 8.85 (dd, 1H, $^{3}J_{H-H}$ = 8.1 Hz, $^{4}J_{H-H}$ = 1.5 Hz), 8.82 (d, 1H, $^{3}J_{H-H}$ = 8.1 Hz), 8.60-8.58 (m, 4H), 8.20-8.16 (m, 1H), 8.07-8.03 (m, 1H), 6.84-6.79 (m, 4H), 5.39-5.32 (m, 4H), 3.34-3.16 (m, 8H), 1.89-1.81 (m, 8H), 1.66-1.55 (m, 8H), 1.44-1.24 (m, 32H), 1.11 (s, 9H), 1.10 (s, 9H), 0.92-0.85 (m, 12H); **^{13}C NMR** ($CDCl_3$, 125 MHz): δ 153.5, 153.4, 152.0, 151.1, 150.3, 150.2, 149.3, 144.8, 143.9, 141.1, 140.7, 134.2, 132.4, 131.6, 130.8, 130.1, 128.8, 128.4, 126.4, 126.2, 125.9, 123.7, 123.2, 122.4, 119.8, 119.5, 117.9, 117.8, 93.7, 91.6, 34.1, 33.8, 32.0, 31.4, 29.4, 29.3, 28.6, 22.8, 14.2; **^{11}B NMR** ($CDCl_3$, 96 MHz) δ -14.6; **^{19}F NMR** ($CDCl_3$, 282 MHz): δ -138.0 (m, 4F), -149.0 (m, 4F); **UV-vis** (THF): λ (nm) (log ε/dm^3 mol^{-1} cm^{-1}) = 609 (sh), 581 (5.1), 297 (4.9); **HRMS**

(MALDI-TOF) m/z: [M]+ Calcd for $C_{102}H_{104}B_2F_8N_{12}O_2S_4$ 1830.7369; Found 1830.7365; **Mp** > 250 ºC.

3. NMR Spectra

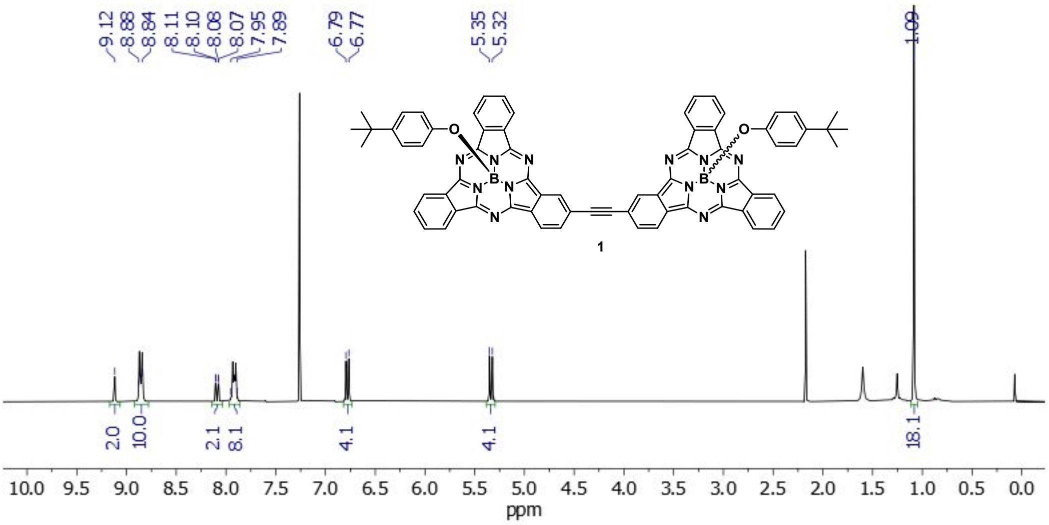

Figure S3.1. ^{1}H-NMR spectrum ($CDCl_3$, 300 MHz) of **1**.

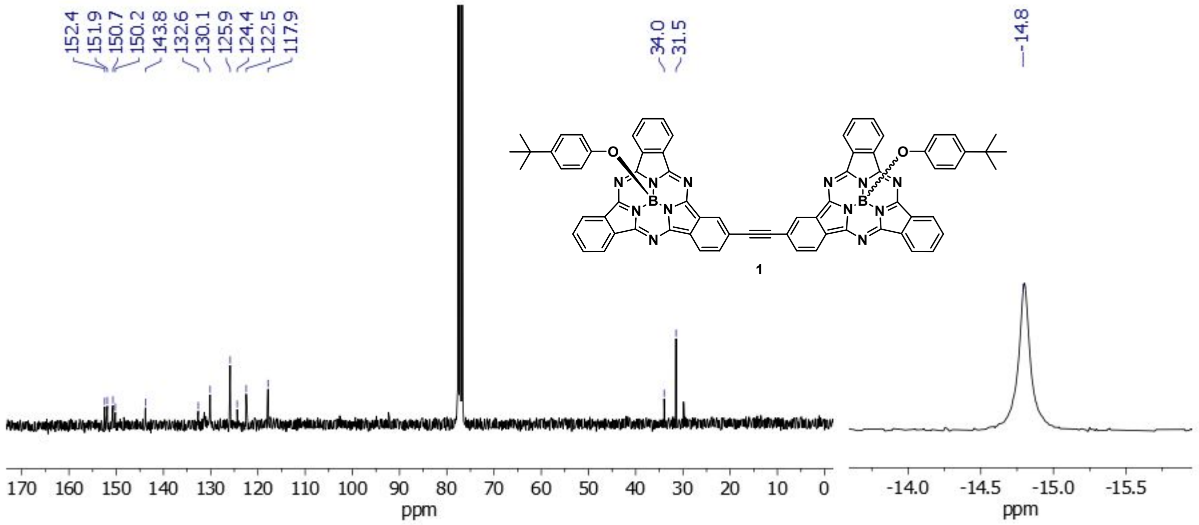

Figure S3.2. ^{13}C-NMR (left; 125 MHz) and ^{11}B-NMR (right; 96 MHz) spectra ($CDCl_3$) of **1**.

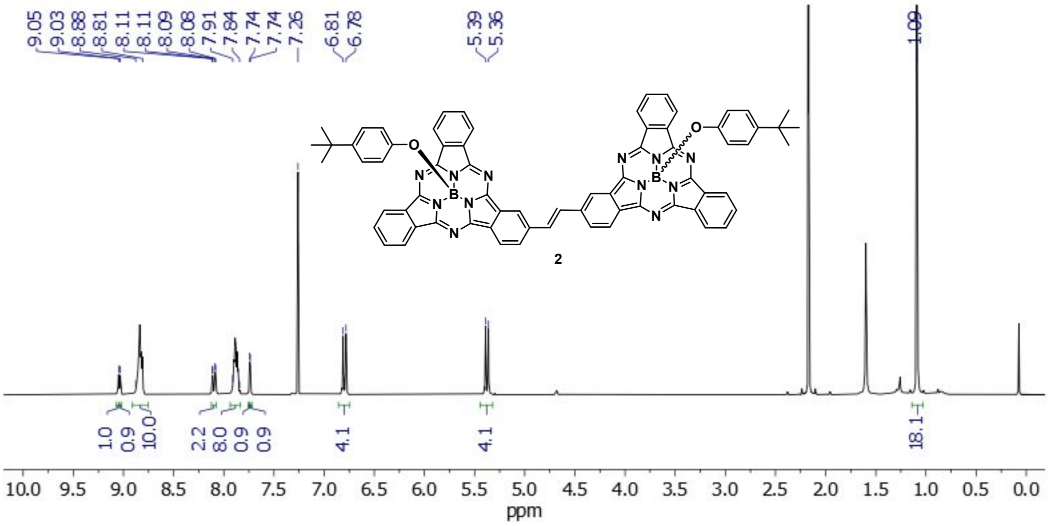

Figure S3.3. ^{1}H-NMR spectrum ($CDCl_3$, 300 MHz) of **2**.

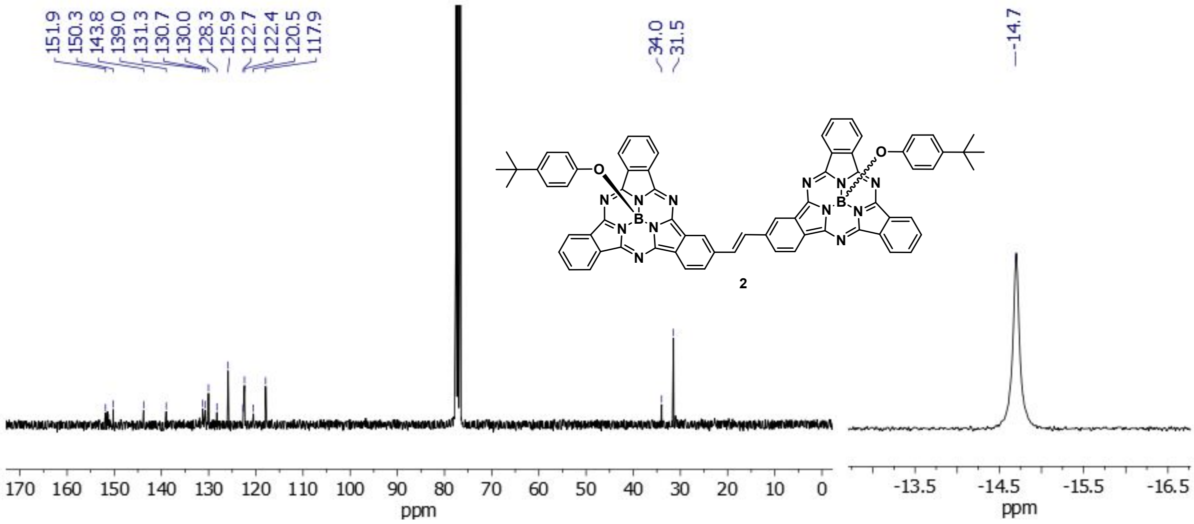

Figure S3.4. ^{13}C-NMR (left; 125 MHz) and ^{11}B-NMR (right; 96 MHz) spectra ($CDCl_3$) of **2**.

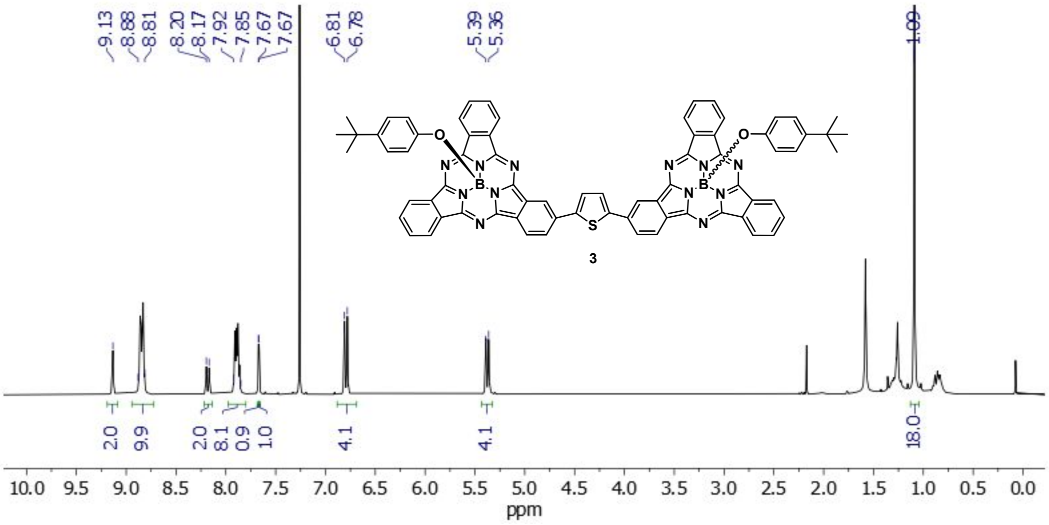

Figure S3.5. ^{1}H-NMR spectrum ($CDCl_3$, 300 MHz) of **3**.

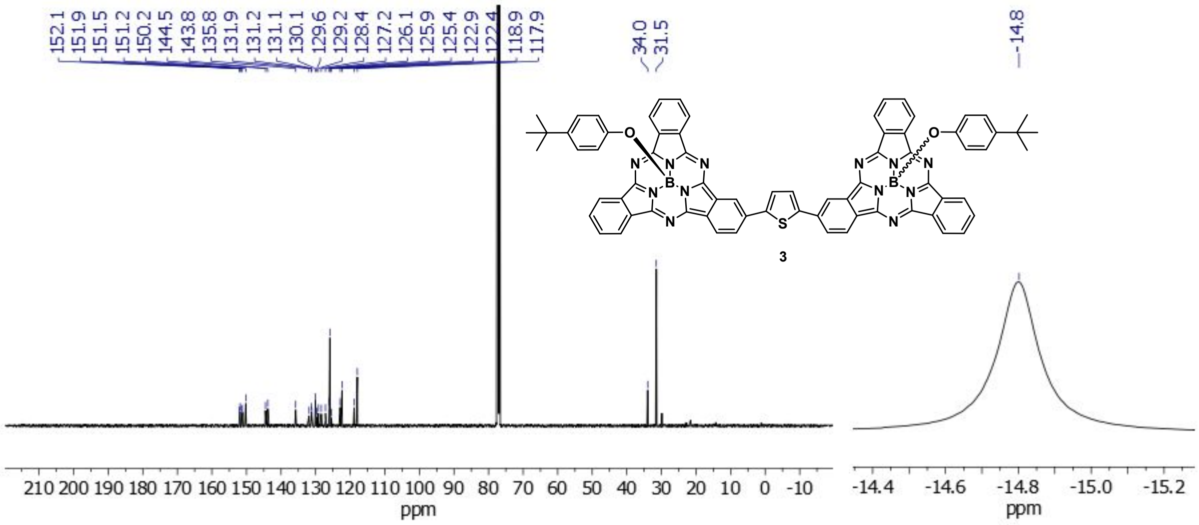

Figure S3.6. ^{13}C-NMR (left; 125 MHz) and ^{11}B-NMR (right; 96 MHz) spectra ($CDCl_3$) of **3**.

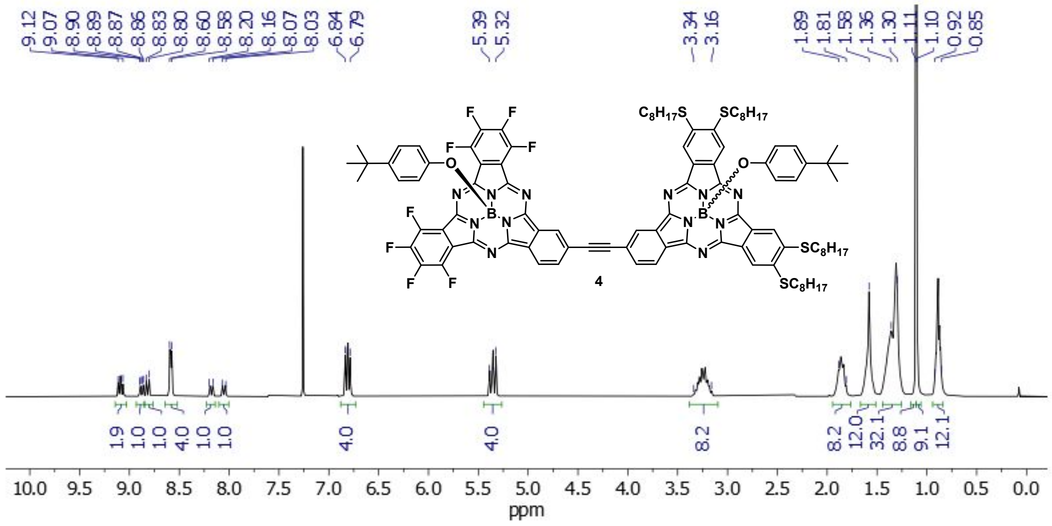

Figure S3.7. ^{1}H-NMR spectrum ($CDCl_3$, 300 MHz) of **4**.

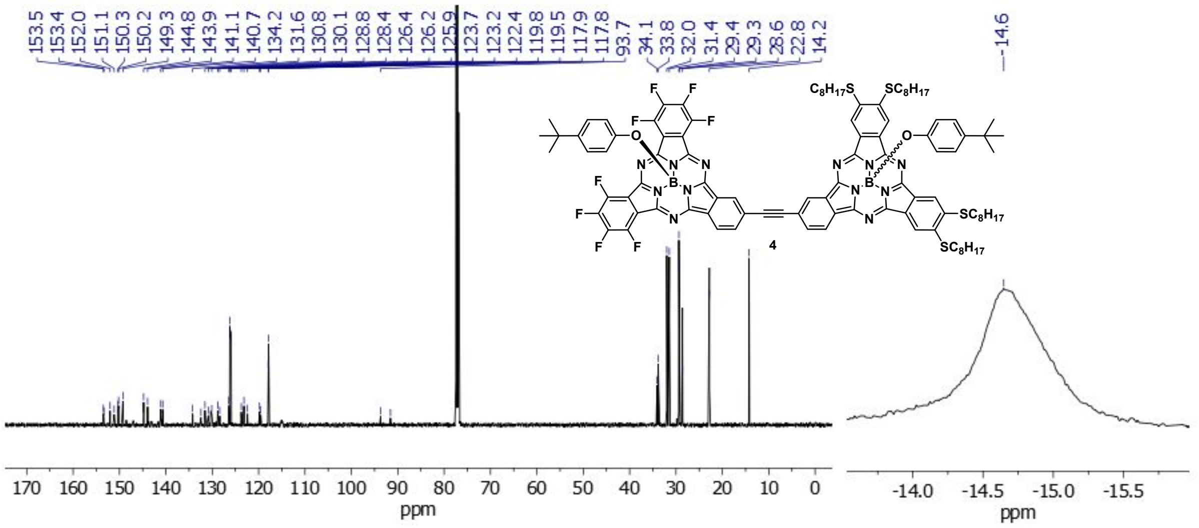

Figure S3.8. ^{13}C-NMR (left; 125 MHz) and ^{11}B-NMR (right; 96 MHz) spectra ($CDCl_3$) of **4**.

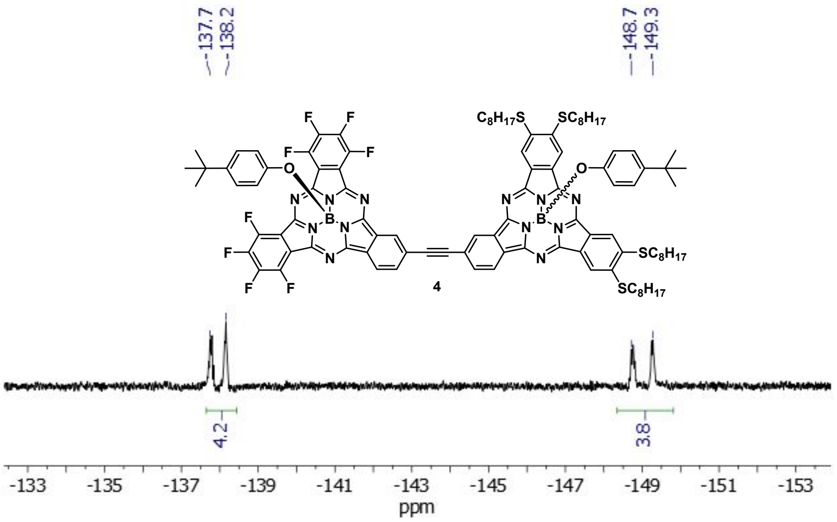

Figure S3.9. ^{19}F-NMR spectrum ($CDCl_3$, 282 MHz) of **4**.

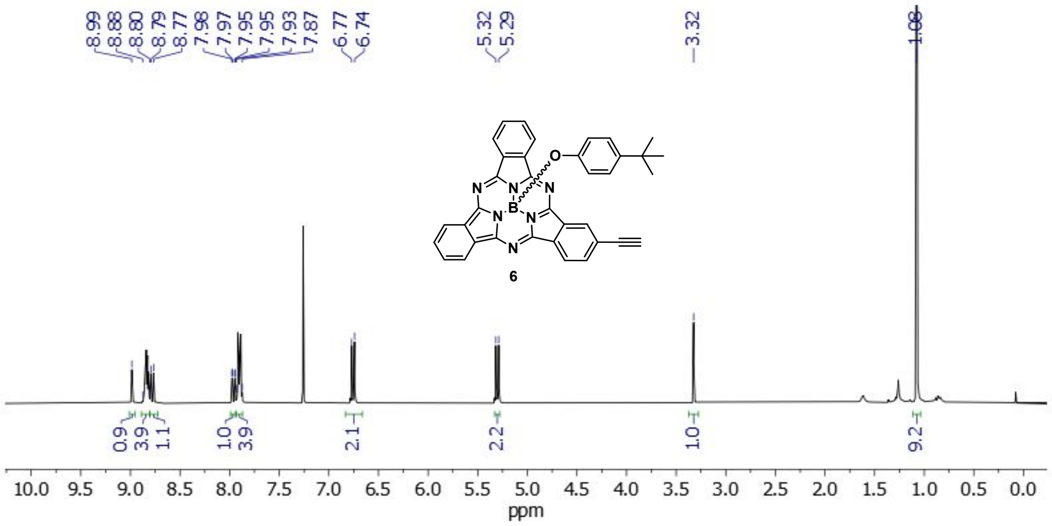

Figure S3.10. ^{1}H-NMR spectrum ($CDCl_3$, 300 MHz) of **6**.

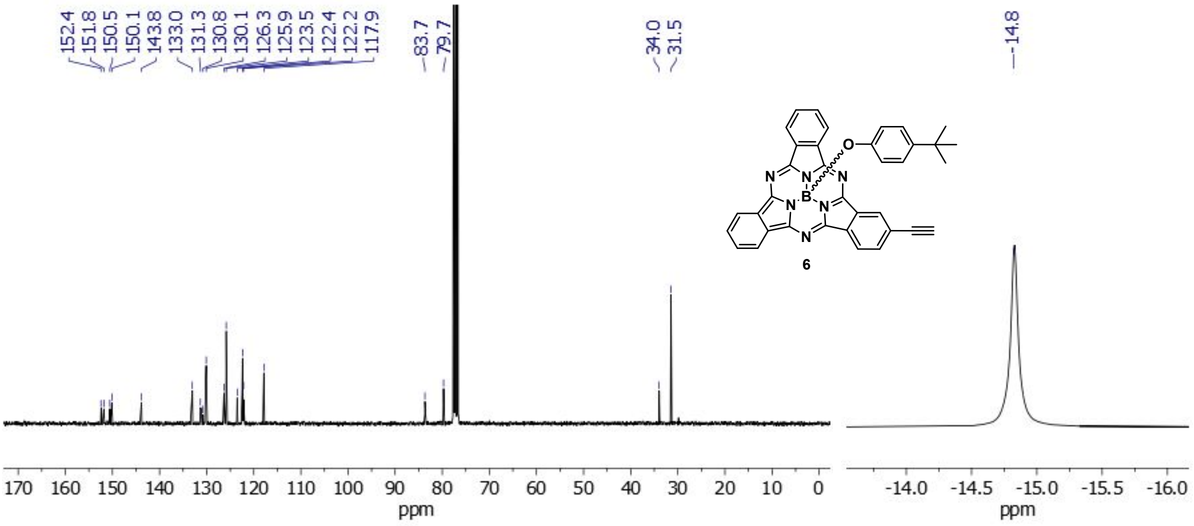

Figure S3.11. ^{13}C-NMR (left; 125 MHz) and ^{11}B-NMR (right; 96 MHz) spectra ($CDCl_3$) of **6**.

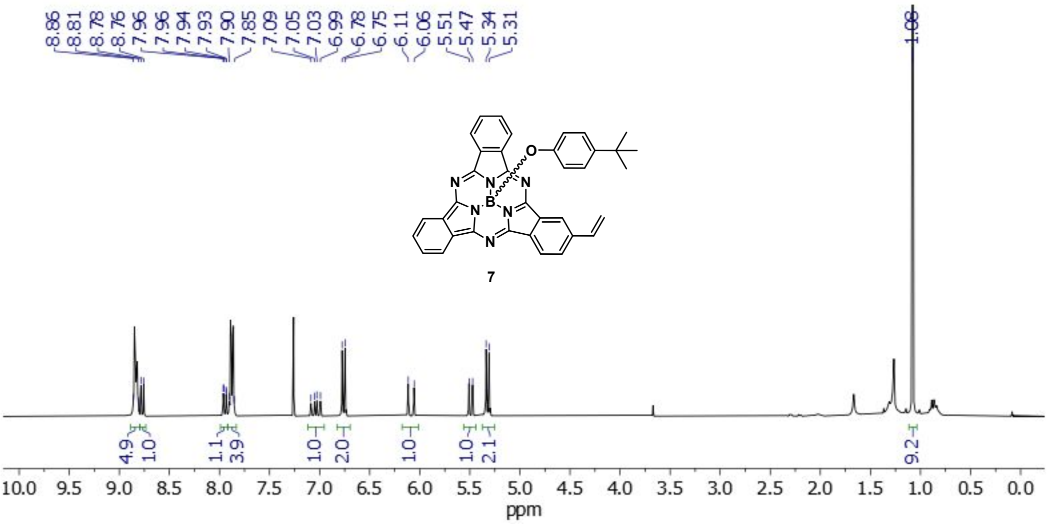

Figure S3.12. ^{1}H-NMR spectrum ($CDCl_3$, 300 MHz) of **7**.

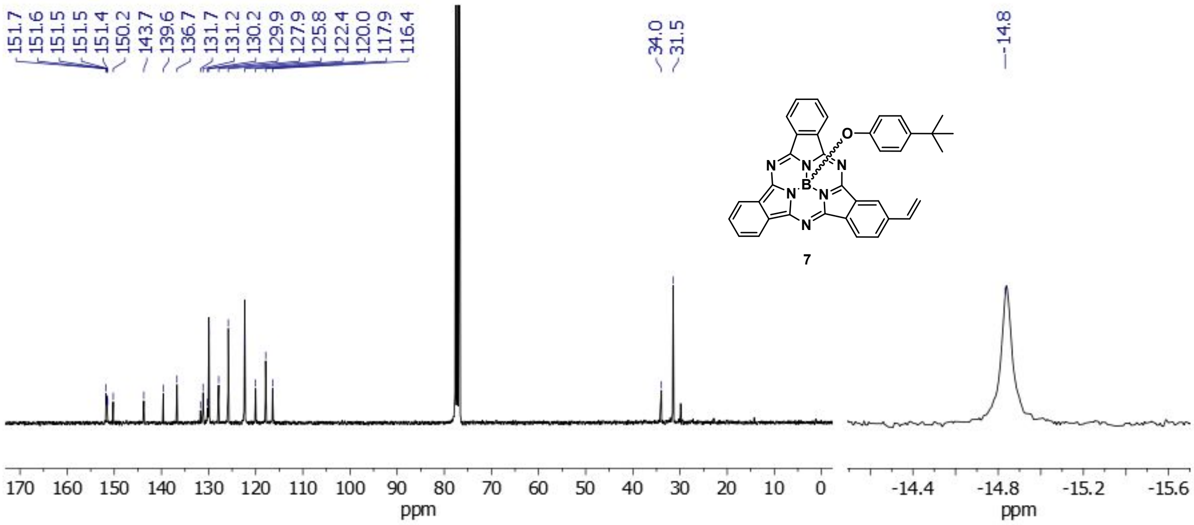

Figure S3.13. ^{13}C-NMR (left; 125 MHz) and ^{11}B-NMR (right; 96 MHz) spectra ($CDCl_3$) of **7**.

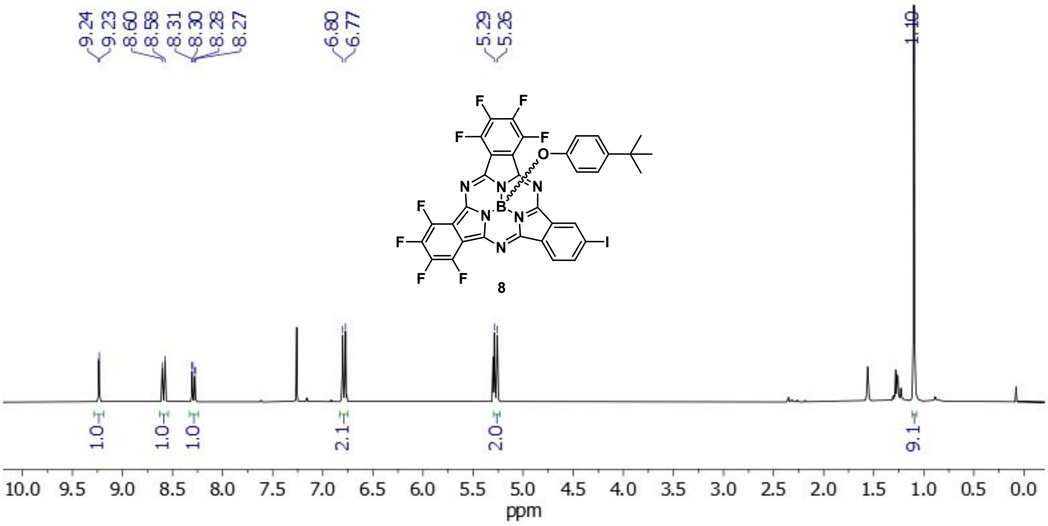

Figure S3.14. ^{1}H-NMR spectrum ($CDCl_3$, 300 MHz) of **8**.

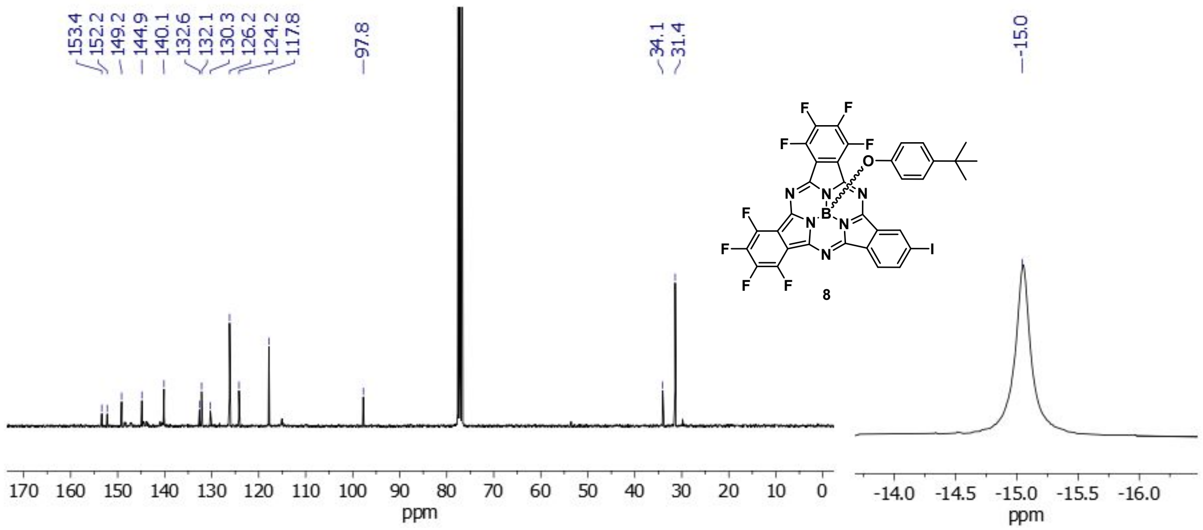

Figure S3.15. ^{13}C-NMR (left; 125 MHz) and ^{11}B-NMR (right; 96 MHz) spectra ($CDCl_3$) of **8**.

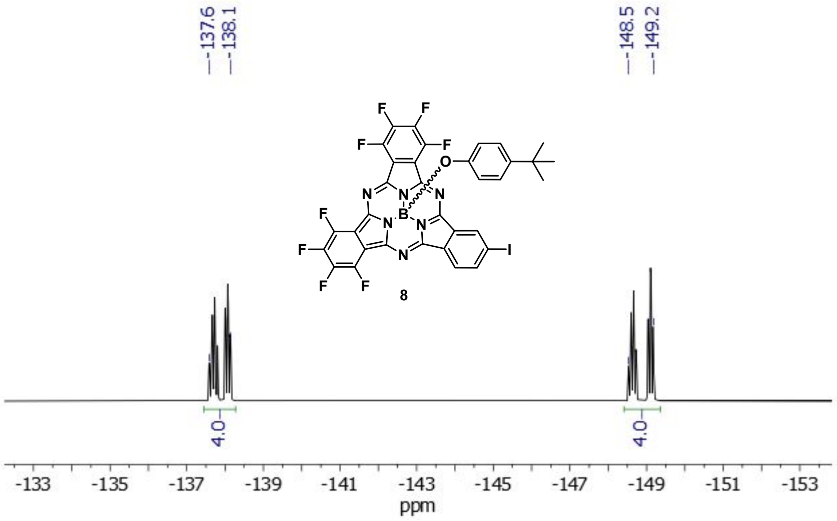

Figure S3.16. ^{19}F-NMR spectrum ($CDCl_3$, 282 MHz) of **8**.

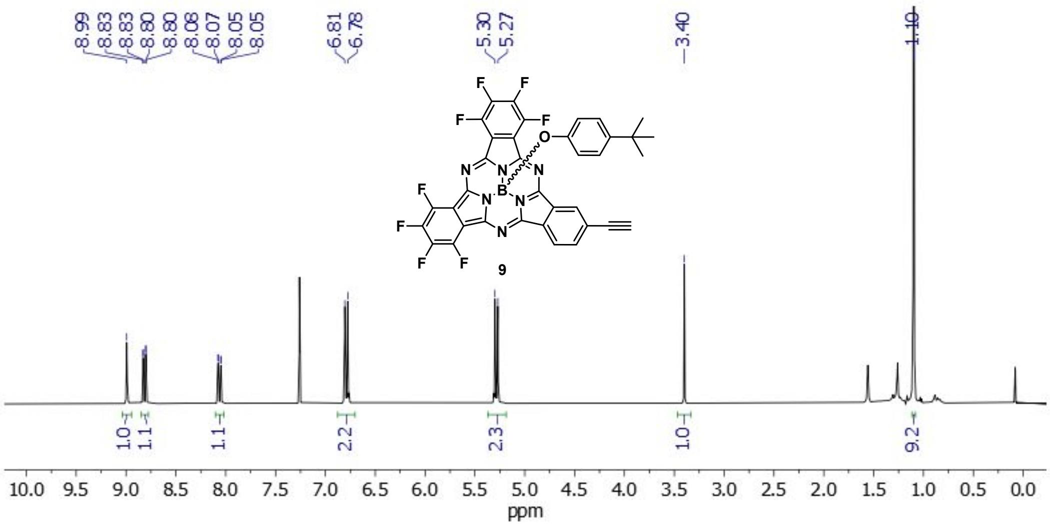

Figure S3.17. ^{1}H-NMR spectrum ($CDCl_3$, 300 MHz) of **9**.

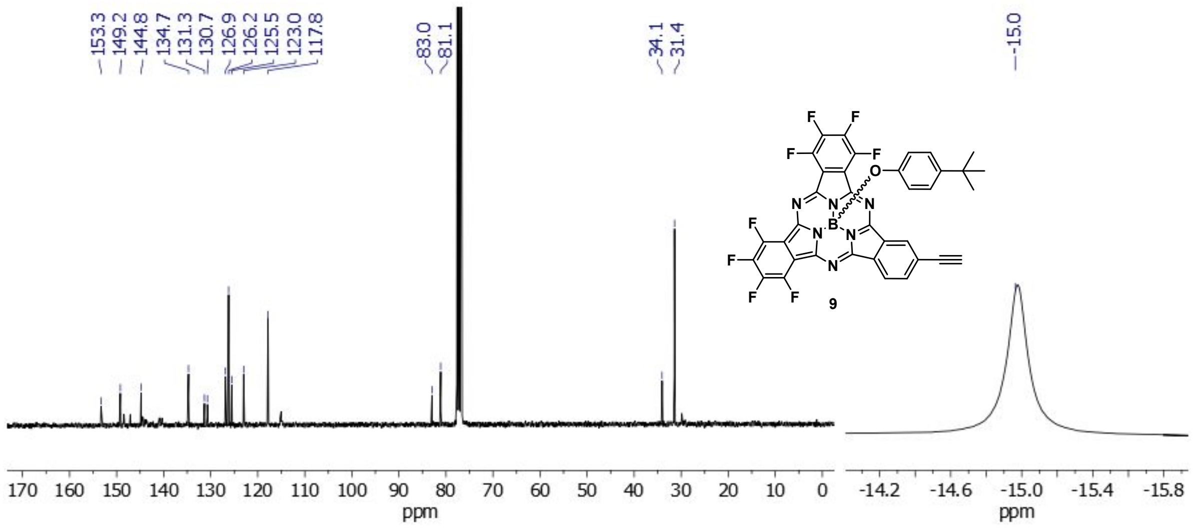

Figure S3.18. ^{13}C-NMR (left; 125 MHz) and ^{11}B-NMR (right; 96 MHz) spectra ($CDCl_3$) of **9**.

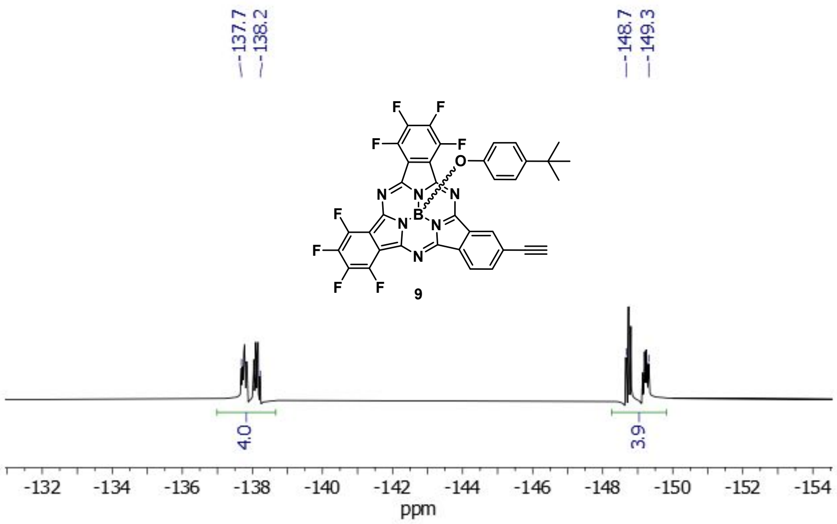

Figure S3.19. ^{19}F-NMR spectrum ($CDCl_3$, 282 MHz) of **9**.

4. Mass Spectra

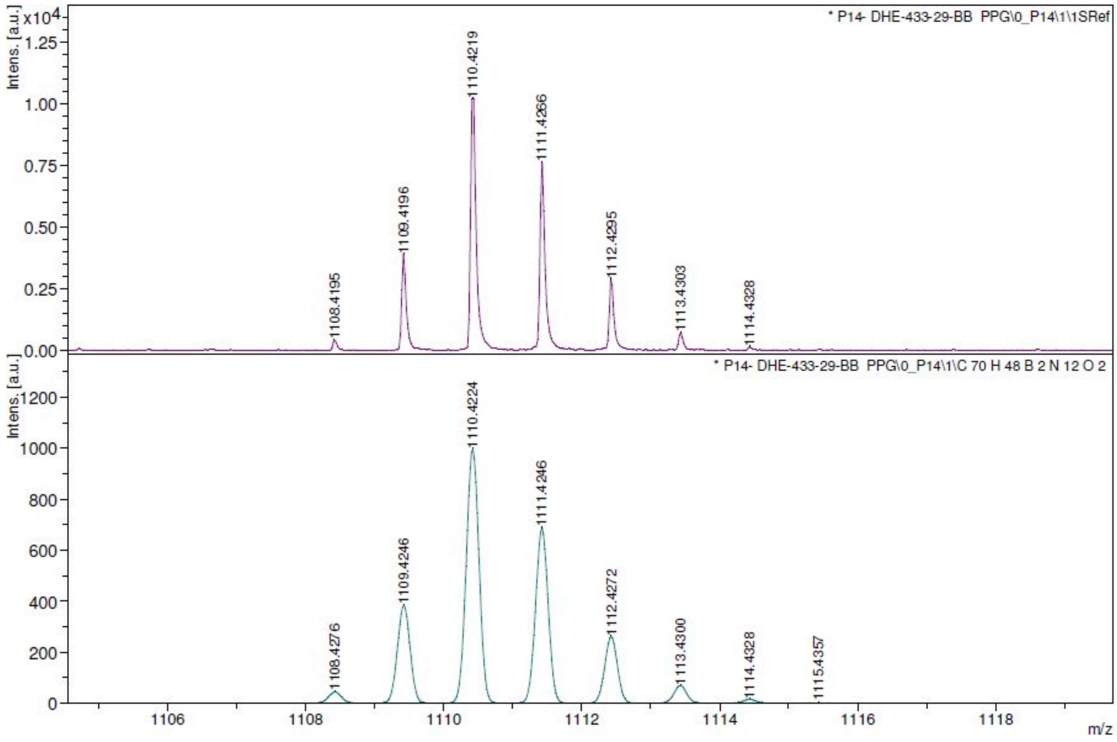

Figure S4.1. MALDI-TOF mass spectrum of **1**.

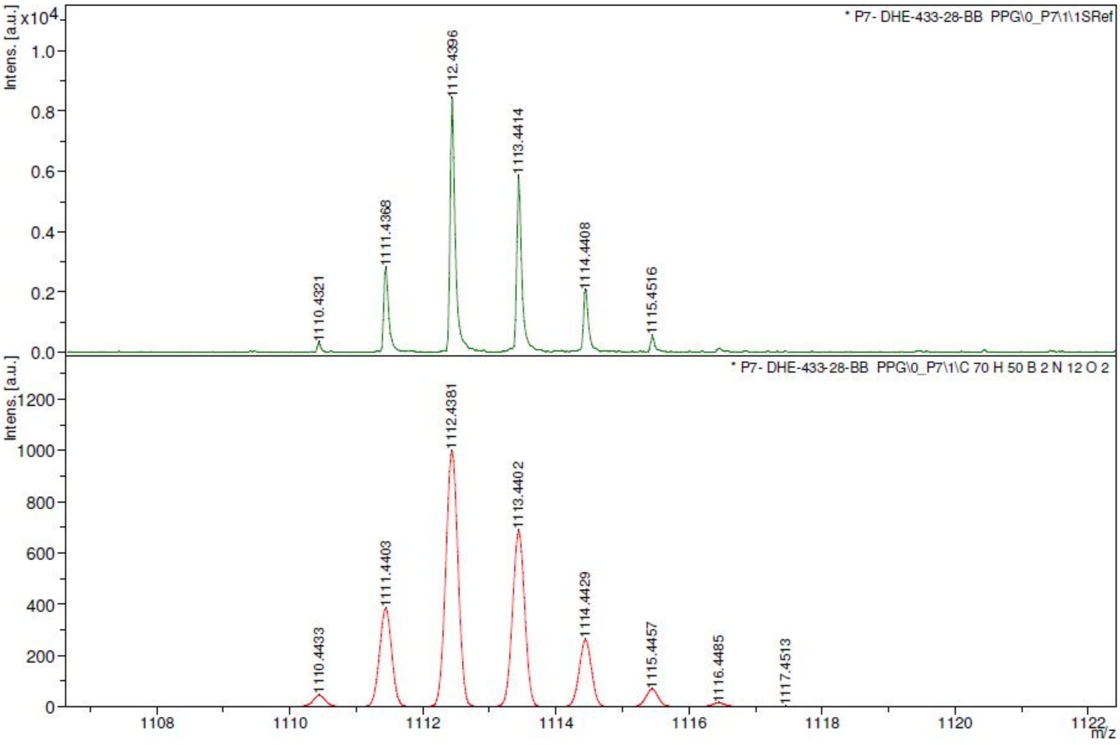

Figure S4.2. MALDI-TOF mass spectrum of **2**.

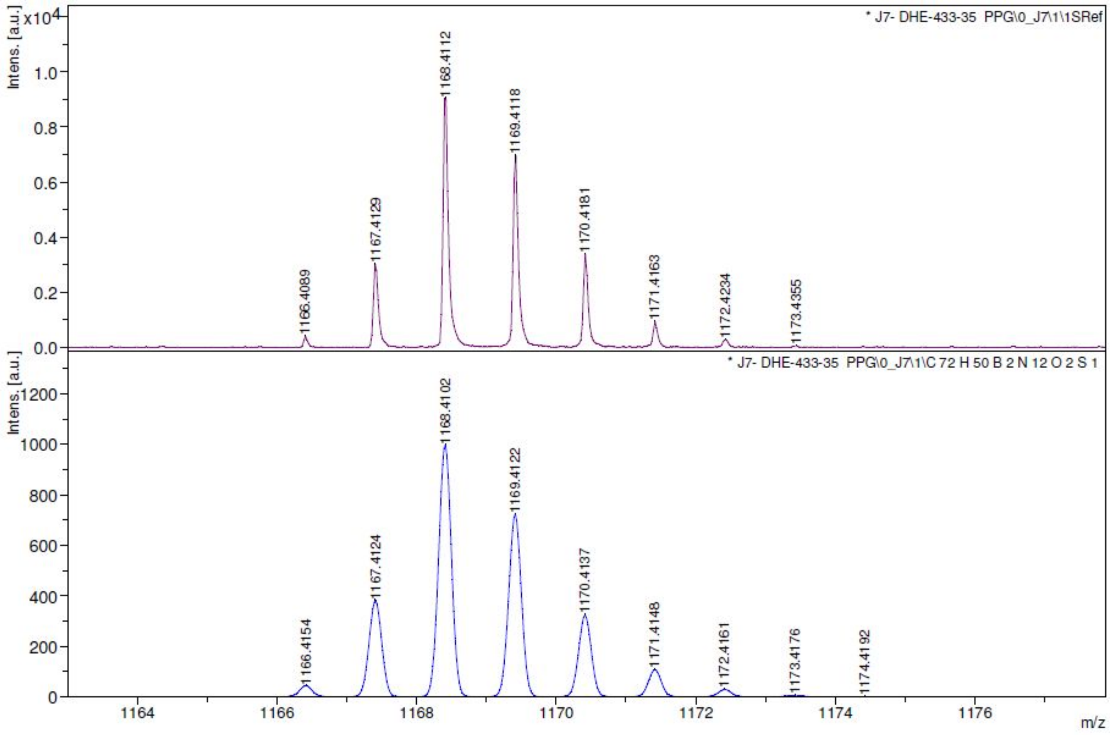

Figure S4.3. MALDI-TOF mass spectrum of **3**.

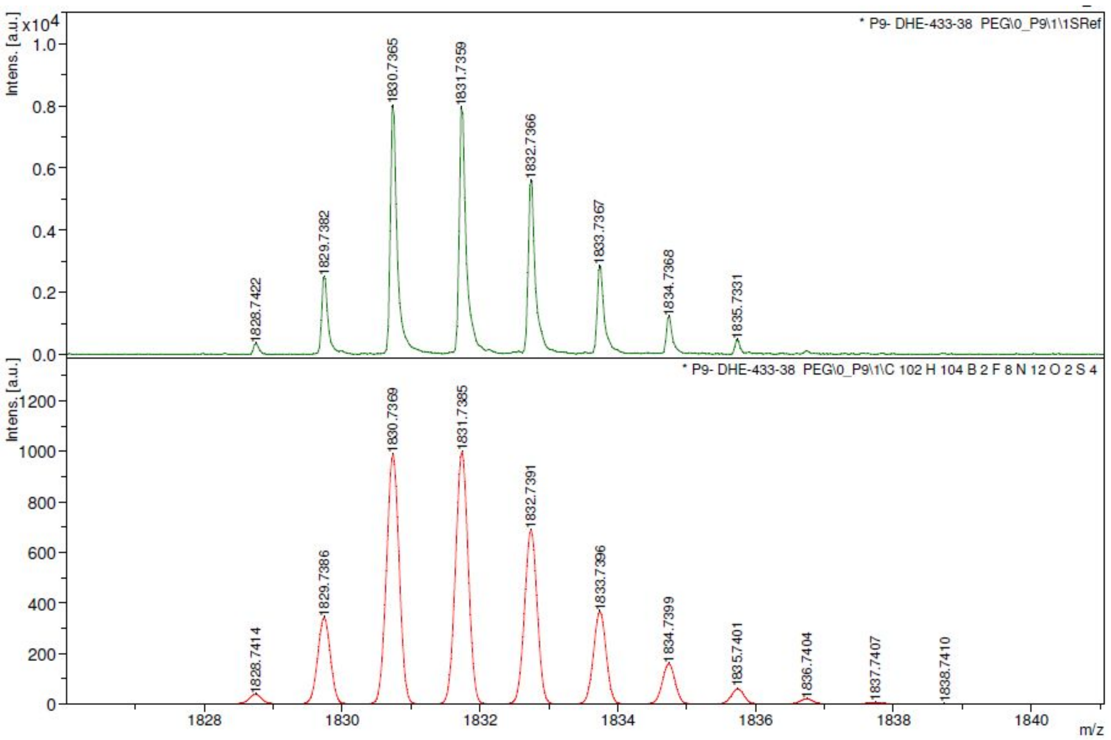

Figure S4.4. MALDI-TOF mass spectrum of **4**.

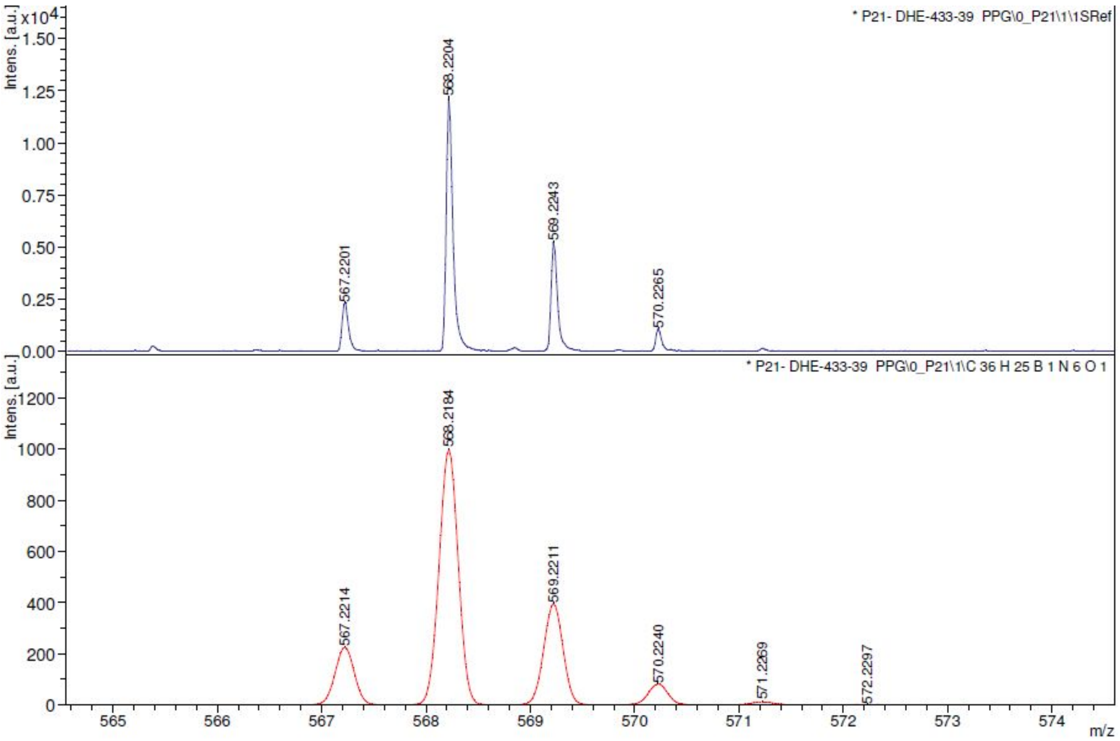

Figure S4.5. MALDI-TOF mass spectrum of **6**.

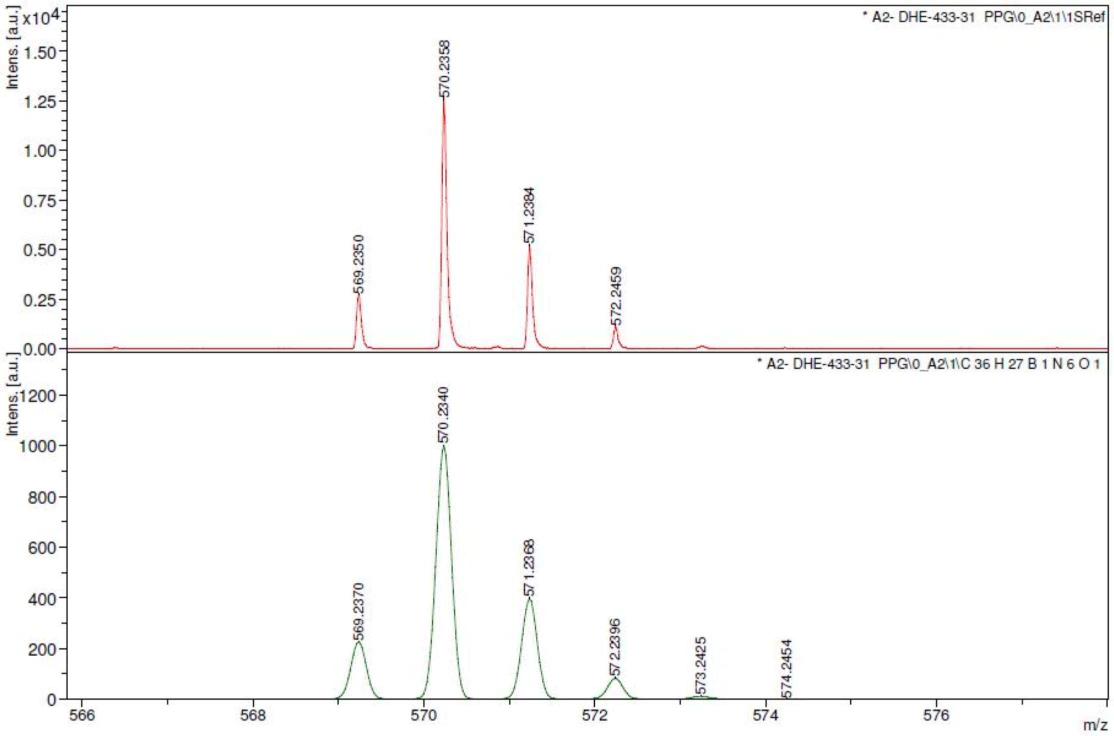

Figure S4.6. MALDI-TOF mass spectrum of **7**.

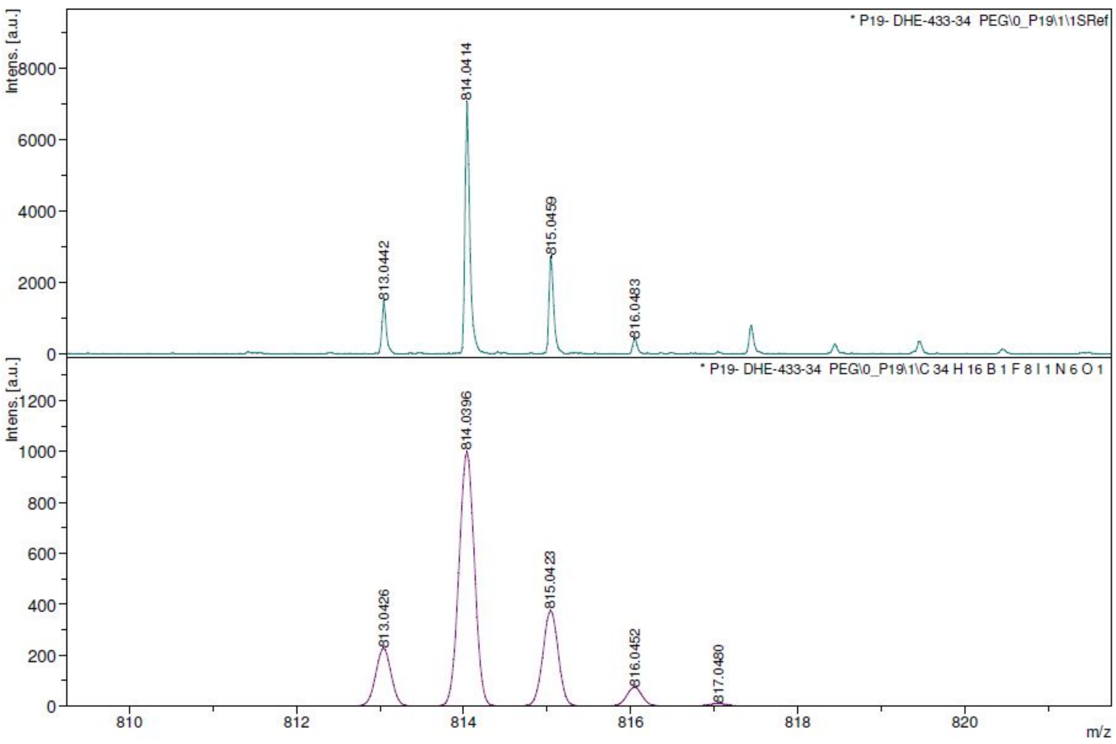

Figure S4.7. MALDI-TOF mass spectrum of **8.**

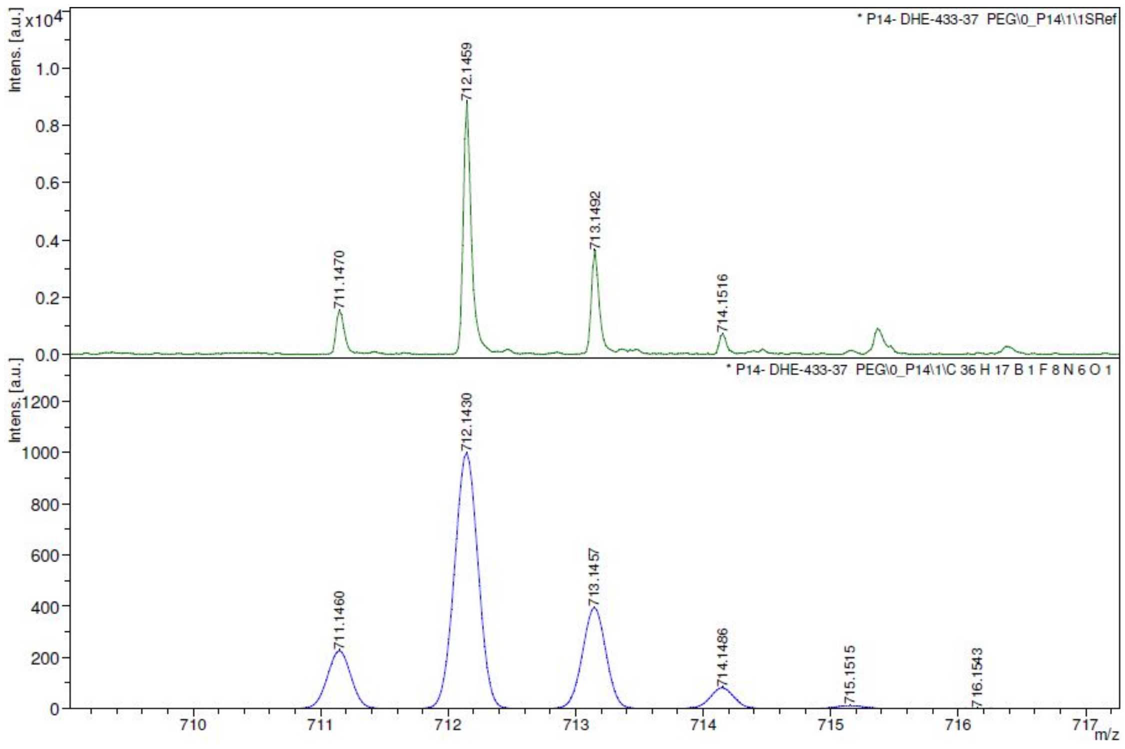

Figure S4.**8**. MALDI-TOF mass spectrum of **9**.

5. Computational Studies

All the reported structures were optimized at Density Functional Theory (DFT) level, using the B3LYP functional and the 6-31G(d,p) calculation base,[8,9,10] considering the solvent (tetrahydrofuran, THF) through the Polarizable Continuum Model (PCM).[11,12] The analytical harmonic frequencies were calculated at the same theoretical level to confirm the nature of the stationary points. Time-dependent DFT (TD-DFT) calculations were carried out at the CAM-B3LYP/6-31+G(d,p) level.[13] All these theoretical calculations were carried out using methods implemented in the Gaussian 16 package.[14] To save computational costs, octyl- groups were replaced by methyl groups.

TD-DFT calculations

Excited state	Energy (nm)	*f*[a]	Orbitals[b] (coefficient)
S_1	540	0.9623	H→L (56%)<br>H-1→L+3 (35%)
S_2	508	0.5841	H-1→L (51%)<br>H→L+3 (42%)
S_3	502	0.4076	H-1→L+1 (42%)<br>H→L+2 (50%)
S_4	499	0.3689	H-1→L+2 (47%)<br>H→L+1 (46%)
S_5	360	0.7030	H-4→L (25%)<br>H-2→L (30%)<br>H-1→L+3 (38%)<br>H→L (28%)
S_6	350	0.0003	H-1→L (45%)<br>H→L+3 (43%)
S_7	327	0.0172	H-1→L+2 (43%)<br>H→L+1 (37%)
S_8	326	0.1849	H-1→L+1 (44%)<br>H→L+2 (45%)

Table S5.1. Selected transition properties of **1** calculated at CAM-B3LYP/6-31+G(d,p) level of theory. [a]Oscillator strength. [b]MOs involved in the transitions (H and L denoting HOMO and LUMO).

Excited state	Energy (nm)	*f*[a]	Orbitals[b] (coefficient)
S_1	544	0.9795	H→L (57%)<br>H-1→L+3 (38%)
S_2	509	0.7146	H-1→L (44%)<br>H→L+2 (25%)<br>H→L+3 (37%)
S_3	504	0.3695	H-1→L (24%)<br>H-1→L+1 (40%)<br>H→L+1 (25%)<br>H→L+2 (34%)<br>H→L+3 (27%)
S_4	502	0.3056	H-1→L+2 (46%)<br>H→L+1 (42%)<br>H→L+2 (25%)

S_5	370	0.7330	H-2→L (43%)<br>H-1→L+3 (37%)<br>H→L (25%)
S_6	356	0.0539	H-1→L (42%)<br>H→L+3 (45%)
S_7	334	0.0725	H-2→L+1 (28%)<br>H-1→L+1 (27%)<br>H-1→L+2 (28%)<br>H→L+1 (37%)
S_8	333	0.1358	H-2→L+2 (28%)<br>H-1→L+1 (34%)<br>H-1→L+2 (23%)<br>H→L+2 (42%)

Table S5.2. Selected transition properties of **2** calculated at CAM-B3LYP/6-31+G(d,p) level of theory. [a]Oscillator strength. [b]MOs involved in the transitions (H and L denoting HOMO and LUMO).

Excited state	Energy (nm)	f[a]	Orbitals[b] (coefficient)
S_1	535	0.6004	H→L (54%)<br>H-1→L+1 (31%)<br>H-1→L+3 (29%)
S_2	509	0.7699	H-1→L (49%)<br>H→L+1 (34%)<br>H→L+3 (33%)
S_3	504	0.5781	H-1→L+1 (34%)<br>H-1→L+3 (33%)<br>H→L+2 (49%)
S_4	501	0.2524	H-1→L+2 (47%)<br>H→L+1 (35%)<br>H→L+3 (35%)
S_5	371	0.8785	H-2→L (48%)<br>H-1→L+3 (26%)
S_6	351	0.0006	H-2→L+1 (26%)<br>H-1→L (41%)<br>H→L+1 (34%)<br>H→L+3 (29%)
S_7	333	0.0227	H-2→L+1 (35%)<br>H-1→L+2 (34%)<br>H-1→L+3 (37%)
S_8	332	0.1560	H-2→L+2 (33%)<br>H-1→L+1 (34%)<br>H→L+2 (43%)

Table S5.3. Selected transition properties of **3** calculated at CAM-B3LYP/6-31+G(d,p) level of theory. [a]Oscillator strength. [b]MOs involved in the transitions (H and L denoting HOMO and LUMO).

Excited state	Energy (nm)	f[a]	Orbitals[b] (coefficient)
S_1	544	0.6753	H-1→L (31%)<br>H→L (34%)<br>H→L+3 (41%)
S_2	520	0.7327	H-1→L (48%)<br>H-1→L+1 (40%)
S_3	518	0.7971	H→L+2 (66%)
S_4	506	0.3111	H-1→L (26%)<br>H-1→L+1 (50%)<br>H→L+3 (35%)
S_5	378	0.3621	H→L (47%)<br>H→L+3 (29%)
S_6	361	0.0034	H→L (30%)<br>H→L+1 (58%)
S_7	350	0.6349	H-1→L+3 (28%)
S_8	344	0.0911	H-2→L+2 (56%)

Table S5.4. Selected transition properties of **4** calculated at CAM-B3LYP/6-31+G(d,p) level of theory. [a]Oscillator strength. [b]MOs involved in the transitions (H and L denoting HOMO and LUMO).

Calculated UV-vis espectra

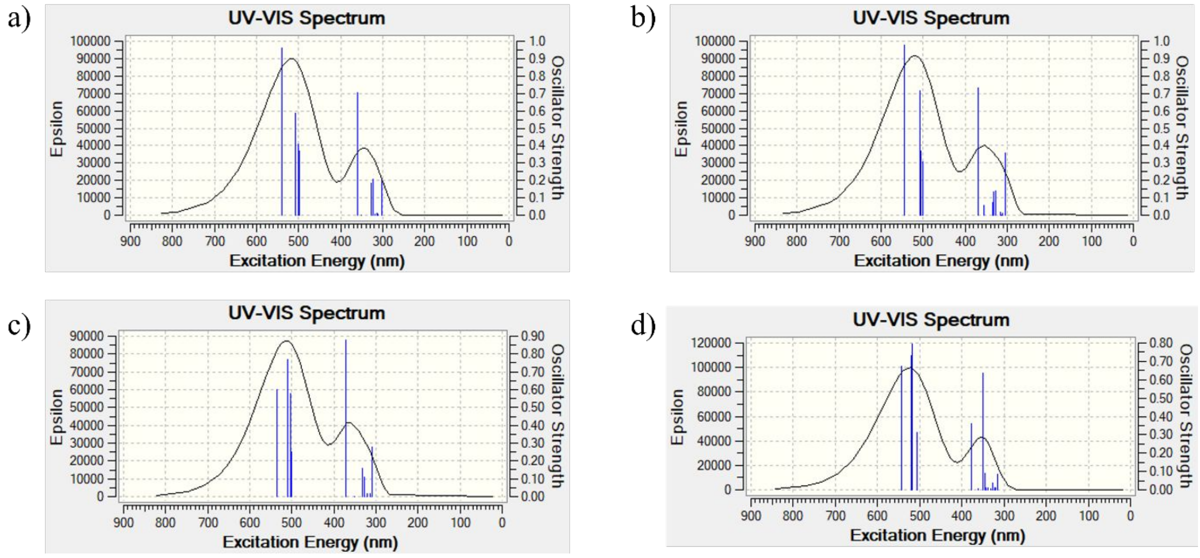

Figure S5.1. Calculated spectra of **1** (a), **2** (b), **3** (c) and **4** (d), calculated at CAM-B3LYP/6-31+G(d,p) level of theory.

Selected molecular orbitals and their energy levels

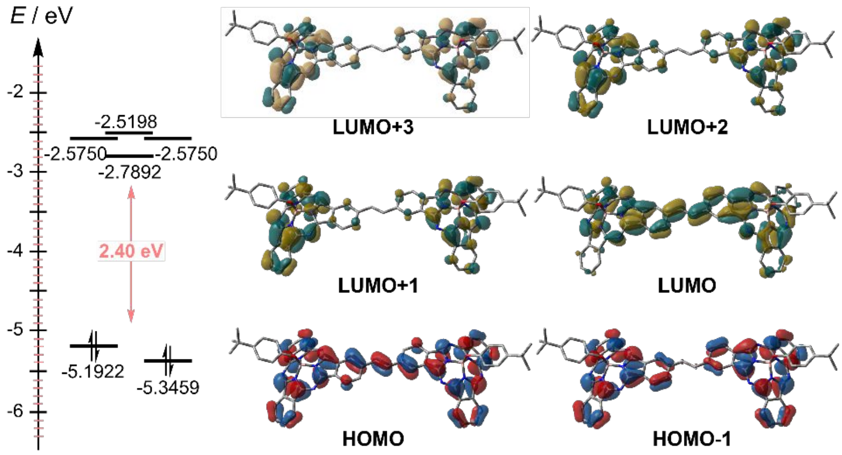

Figure S5.2. Selected molecular orbitals of **2** and their energy levels calculated by DFT at the B3LYP/6-31G(d,p) level of theory. Hydrogen atoms are omitted for clarity.

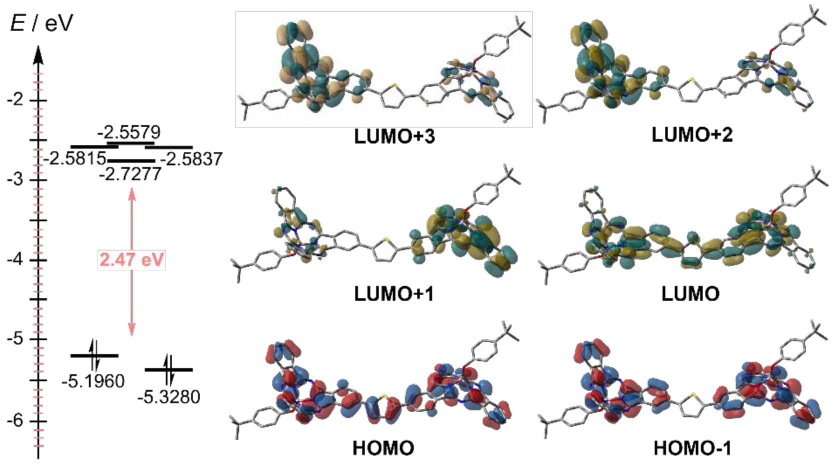

Figure S5.3. Selected molecular orbitals of **3** and their energy levels calculated by DFT at the B3LYP/6-31G(d,p) level of theory. Hydrogen atoms are omitted for clarity.

<u>Electrostatic potential (ESP) map of 4</u>

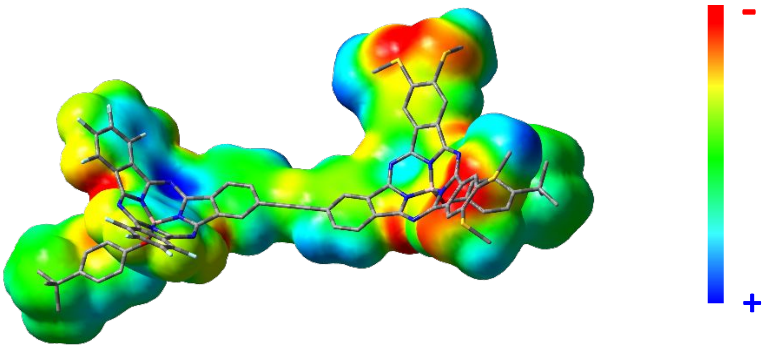

Figure S5.4. ESP map of **4** simulated by DFT calculations (B3LYP/6-31G(d,p)). Hydrogen atoms are omitted for clarity.

6. Analytical HPLC Resolution of Racemic SubPc Dimers 1, 2, 3 and 4

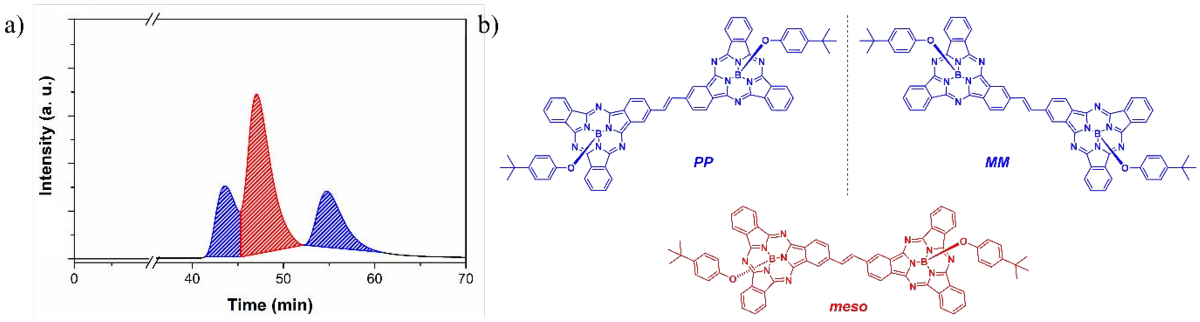

Figure S6.1. HPLC chromatogram (a) of racemic **2** with peaks corresponding to the chiral species (two enantiomers; blue trace) and *meso* compound (red trace) (b). The percentage area underneath the first, second and third peak is 25.0 %, 50.0 %, and 25.0 % respectively. HPLC (CHIRALPAK® IC, dichloromethane/n-hexane = 80/20, flow rate = 1.0 mL/min, temperature = 20 ºC, λ = 595 nm) t_R = 43.6 min (blue), 47.0 min (red), 54.7 min (blue).

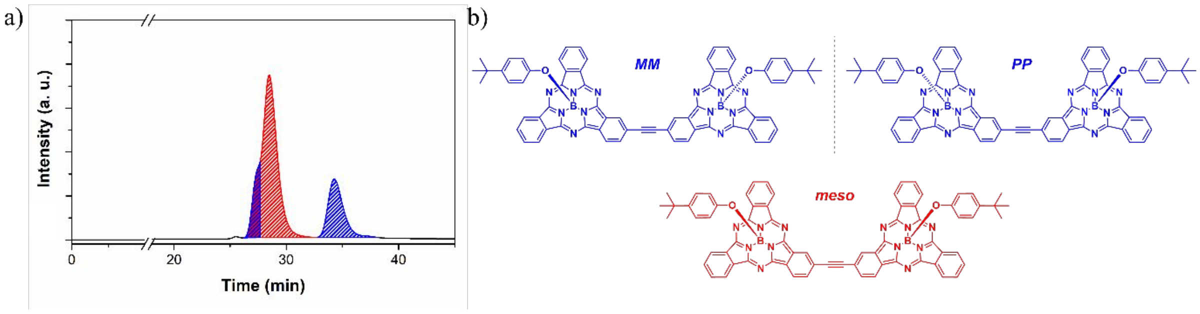

Figure S6.2. HPLC chromatogram (a) of racemic dimer **1** with peaks corresponding to the chiral species (two enantiomers; blue trace) and *meso* compound (red trace) (b). The percentage area underneath the first and second peak is 75.0 %, and 25.0 % respectively. HPLC (CHIRALPAK® IC, dichloromethane, flow rate = 1.0 mL/min, temperature = 20 ºC, λ = 595 nm) t_R = 27.4 min (blue shoulder), 28.5 min (red), 34.3 min (blue).

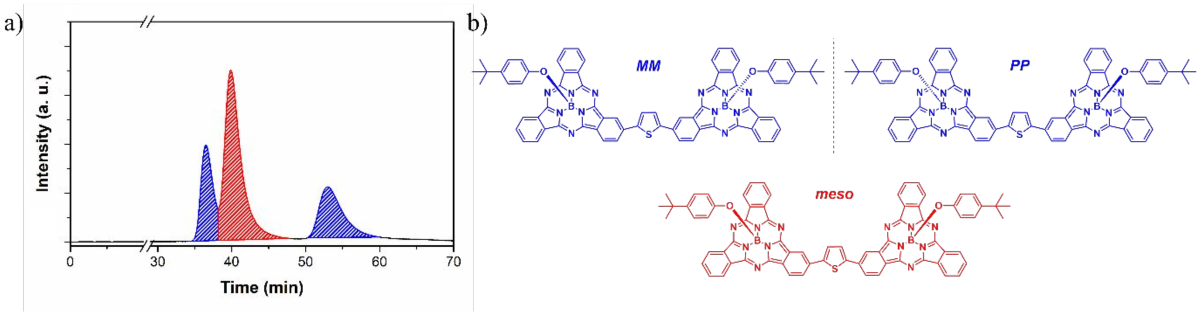

Figure S6.3. HPLC chromatogram (a) of racemic dimer **3** with peaks corresponding to the chiral species (two enantiomers; blue trace) and *meso* compound (red trace) (b). The percentage area underneath the first, second and third peak is 25.0 %, 50.0 %, and 25.0 % respectively. HPLC (CHIRALPAK® IC, dichloromethane/n-hexane = 80/20, flow rate = 1.0 mL/min, temperature = 20 ºC, λ = 595 nm) t_R = 36.5 min (blue), 39.9 min (red), 53.0 min (blue).

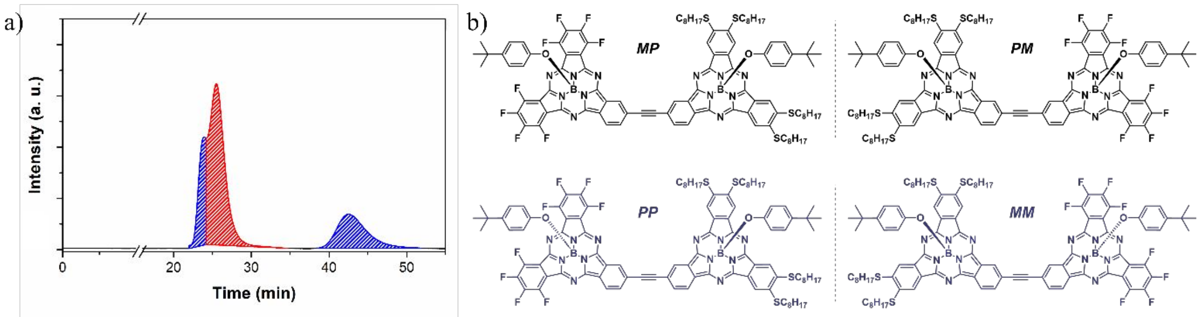

Figure S6.4. HPLC chromatogram (a) of racemic dimer **4** with colors corresponding to the two different diasterioisomeric pair of molecules (b). The structural nature of each eluted species was not determined. The percentage area underneath the first, and second peak is 75.0 %, and 25.0 % respectively. HPLC (CHIRALPAK® IC, dichloromethane/n-hexane = 80/20, flow rate = 1.0 mL/min, temperature = 20 ºC, λ = 595 nm) t_R = 23.9 min (blue shoulder), 25.5 min (red), 42.5 min (blue).

7. Supporting References

[1] N. R. Babij, E. O. McCusker, G. T. Whiteker, B. Canturk, N. Choy, L. C. Creemer, C. V. D. Amicis, N. M. Hewlett, P. L. Johnson, J. A. Knobelsdorf, F. Li, B. A. Lorsbach, B. M. Nugent, S. J. Ryan, M. R. Smith and Q. Yang, *Org. Process Res. Dev.*, **2016**, *20*, 661.

[2] J. R. Lakowicz, in Principles of fluorescence spectroscopy, 3rd ed. Springer, New York, **2006**.

[3] C. Romero-Nieto, J. Guilleme, C. Villegas, J. L. Delgado, D. González-Rodríguez, N. Martin, T. Torres, D. M. Guldi, J. Mater. Chem. **2011**, *21*, 15914.

[4] Mauriccio, M.S.; Polina, I.; Greenberg, S.; Lever, A. B. P.; Leznoff, C. C.; Tomer, B. *Can. J. Chem.* **1985**, *63*, 3057.

[5] del Rey, B.; Keller, U.; Torres, T.; Rojo, G.; Agulló-Lopez, F.; Nonell, S.; Marti, C.; Brasselet, S.; Ledoux, I.; Zyss, J. *J. Am. Chem. Soc.* **1998**, *120*, 12808.

[6] Muñoz, A. V.; Gotfredsen, H.; Jevric, M.; Kadziola, A.; Hammerich, O.; Nielsen, M. B. *J. Org. Chem.* **2018**, *83*, 2227.

[7] Zango, G.; Krug, M.; Krishna, S.; Marinas, V.; Clark, T.; Martinez-Diaz, M.; Guldi, D. M.; Torres, T. *Chem. Sci.* **2020**, *11*, 3448.

[8] Lee, C.; Yang, W.; Parr, R. G. *Phys. Rev. B* **1988**, *37*, 785.

[9] Becke, A. D. *J. Chem. Phys.* **1993**, *98*, 5648.

[10] Kohn, W.; Becke, A. D.; Parr, R. G. *J. Phys. Chem.* **1996**, *100*, 12974.

[11] J. Tomasi, B. Mennucci, R. Cammi, *Chem. Rev.* **2005**, *105*, 2999.

[12] S. Miertus, E. Scrocco, J. Tomasi, *Chem. Phys.* **1981**, *55*, 117.

[13] Yanai, T.; Tew, D. P.; Handy, N. C. *Chem. Phys. Lett.* **2004**, *393*, 51.

[14] Gaussian 16, Revision C.01; Frisch, M. J.; Trucks, G. W.; Schlegel, H. B.; Scuseria, G. E.; Robb, M. A.; Cheeseman, J. R.; Scalmani, G.; Barone, V.; Petersson, G. A.; Nakatsuji, H.; Li, X.; Caricato, M.; Marenich, A. V.; Bloino, J.; Janesko, B. G.; Gomperts, R.; Mennucci, B.; Hratchian, H. P.; Ortiz, J. V.; Izmaylov, A. F.; Sonnenberg, J. L.; Williams-Young, D.; Ding, F.; Lipparini, F.; Egidi, F.; Goings, J.; Peng, B.; Petrone, A.; Henderson, T.; Ranasinghe, D.; Zakrzewski, V. G.; Gao, J.; Rega, N.; Zheng, G.; Liang, W.; Hada, M.; Ehara, M.; Toyota, K.; Fukuda, R.; Hasegawa, J.; Ishida, M.; Nakajima, T.; Honda, Y.; Kitao, O.; Nakai, H.; Vreven, T.; Throssell, K.; Montgomery, J. A., Jr.; Peralta, J. E.; Ogliaro, F.; Bearpark, M. J.; Heyd, J. J.; Brothers, E. N.; Kudin, K. N.; Staroverov, V. N.; Keith, T. A.; Kobayashi, R.; Normand, J.; Raghavachari, K.; Rendell, A. P.; Burant, J. C.; Iyengar, S. S.; Tomasi, J.; Cossi, M.; Millam, J. M.; Klene, M.; Adamo, C.; Cammi, R.; Ochterski, J. W.; Martin, R. L.; Morokuma, K.; Farkas, O.; Foresman, J. B.; Fox, D. J. Gaussian, Inc., Wallingford CT, 2016.
