# Supplementary material for: β-to-β Singly Linked Subphthalocyanine Dimers with Effective π-Conjugation
Source: Org Lett. 2024 Oct 31;26(44):9471–5. doi: 10.1021/acs.orglett.4c03407 (PMC11555671; doi:10.1021/acs.orglett.4c03407)
Supplement: Supplementary file 2 — ol4c03407_si_002.pdf [file ol4c03407_si_002.pdf]

DFT Cartesian Coordinates

β-to-β Singly-Linked Subphthalocyanine Dimers with Effective π-Conjugation

Daniel Holgado, ‡ [a] Marta Gómez-Gómez,‡[a] Jorge Labella,*[a] and Tomás Torres*[a, b, c]

[a] Department of Organic Chemistry, Universidad Autónoma de Madrid, Campus de Cantoblanco, 28049 Madrid, Spain.
[b] Institute for Advanced Research in Chemical Sciences (IAdChem), Universidad Autónoma de Madrid, 28049 Madrid, Spain.
[c] IMDEA-Nanociencia, Campus de Cantoblanco, 28049 Madrid, Spain.

e-mail: jorge.labella@uam.es, tomas.torres@uam.es

‡These authors contributed equally.

Cartesian coordinates

- SubPc **1**

	Coordinates (Angstroms)		
Atom	X	Y	Z
N	-7.28784600	3.04037800	-0.91254000
C	-8.37872700	2.57742900	-0.28504800
C	-6.25392700	2.19466600	-1.06715600
N	-6.34385900	0.86166100	-0.76623500
N	-8.53310300	1.25612200	0.04665800
B	-7.65190900	0.17383000	-0.52017800
C	-5.14055100	0.28725000	-0.46504600
N	-5.03414400	-0.80935300	0.30370500
C	-6.14350000	-1.24096800	0.92301400
N	-7.38136400	-0.71021700	0.66924500
C	-9.37077100	1.06140200	1.11099800
N	-9.29548000	-0.02271700	1.89952900
C	-8.25254100	-0.84823500	1.71564700
C	-9.41145600	3.29109200	0.45769800
C	-10.03154300	2.34258100	1.33155700
C	-4.83442600	2.46249100	-1.22729800
C	-4.13464600	1.26967700	-0.85137600
C	-7.61578100	-1.78147200	2.63742000
C	-6.29607300	-2.02756700	2.14138800
C	-9.77695400	4.63858000	0.49822600
C	-10.77579500	5.02336800	1.38975500
C	-11.38589700	4.09016400	2.24925900
C	-11.01271000	2.74813100	2.23920200
C	-4.12425300	3.61771300	-1.57120400
C	-2.73843100	3.57157400	-1.56654700
C	-2.03427800	2.39173100	-1.19537400
C	-2.74365600	1.23651900	-0.82117700
C	-8.03403000	-2.33529300	3.84960900
C	-7.14277300	-3.15073300	4.54318700

C	-5.84447500	-3.39265800	4.05543300
C	-5.40412100	-2.82553800	2.86172400
H	-11.08797300	6.06229900	1.42710000
H	-9.29706200	5.36204900	-0.15241400
H	-12.15787400	4.42555000	2.93465000
H	-11.47269000	2.03299000	2.91300800
H	-9.02880800	-2.13691400	4.23447600
H	-7.45124000	-3.60540600	5.47931000
H	-5.17425900	-4.02971800	4.62393200
H	-4.39794400	-3.00039500	2.49549900
H	-2.21228200	0.34031600	-0.52138000
H	-2.16628000	4.44948500	-1.84593400
H	-4.64715400	4.52868200	-1.84212000
O	-8.11407800	-0.47722700	-1.71953500
C	-9.29021000	-1.18720600	-1.75782700
C	-9.29320600	-2.56431800	-1.50538500
C	-10.48762200	-0.56054000	-2.10927700
C	-10.47905800	-3.28853800	-1.60033800
H	-8.36186600	-3.05767200	-1.24535400
C	-11.66923300	-1.30093700	-2.20006900
H	-10.48728600	0.50441000	-2.31958000
C	-11.69818100	-2.67957700	-1.94770100
H	-10.44617300	-4.35552900	-1.39986200
H	-12.57726900	-0.77770800	-2.47714300
C	-12.98224600	-3.52459800	-2.04262300
C	-12.79751500	-4.62618400	-3.11334200
H	-11.96213500	-5.29062300	-2.87329400
H	-13.70167500	-5.24095300	-3.18769200
H	-12.60405100	-4.18648000	-4.09741500
C	-13.26681700	-4.18754600	-0.67389300
H	-12.44572600	-4.83815900	-0.35849300
H	-13.41308000	-3.43101100	0.10420500
H	-14.17451000	-4.79914900	-0.72817000
C	-14.21018400	-2.67961500	-2.43074500
H	-14.08646300	-2.20663300	-3.41045300
H	-15.09589700	-3.32087900	-2.48327400
H	-14.41368500	-1.89454800	-1.69510400
N	5.02233300	-0.79618400	0.35130100
C	6.13403500	-1.21569300	0.97470700
C	5.12885200	0.27650400	-0.45046700
N	6.33303500	0.83468900	-0.77782800
N	7.37286200	-0.70009100	0.69576100
B	7.63904300	0.14671900	-0.52139700
C	6.24821100	2.15901300	-1.11636700
N	7.28808300	3.00281100	-0.99476700
C	8.38140000	2.55201000	-0.36274800
N	8.53084900	1.24011300	0.00642200
C	8.25144900	-0.81224400	1.73905100
N	9.30067700	0.01193900	1.89023200
C	9.37597000	1.07178300	1.06937600
C	6.29156000	-1.96643800	2.21487100
C	7.61654500	-1.71369900	2.69315600
C	4.12547500	1.25337800	-0.85688700
C	4.82902200	2.43079400	-1.27217300
C	10.04591400	2.35487200	1.24683300
C	9.42419300	3.28101200	0.35045900

C	5.40051300	-2.73676400	2.96576800
C	5.84681000	-3.27040800	4.17262500
C	7.15033000	-3.02201700	4.64290100
C	8.04097700	-2.23338500	3.91825000
C	2.73459400	1.22963600	-0.81413400
C	2.02893000	2.37864300	-1.21352100
C	2.73685800	3.54351900	-1.62290500
C	4.12282000	3.58059200	-1.64154400
C	11.03675800	2.78095600	2.13432300
C	11.41771400	4.12044900	2.10203900
C	10.80596200	5.03169700	1.22043900
C	9.79771500	4.62691200	0.34856600
H	5.17724300	-3.88580700	4.76522900
H	4.39043000	-2.91623500	2.61277100
H	7.46336500	-3.45021100	5.58993400
H	9.03986100	-2.02958700	4.28940900
H	11.49808400	2.08309700	2.82510700
H	12.19716600	4.47113400	2.77111700
H	11.12430400	6.06941800	1.22481000
H	9.31670200	5.33393300	-0.31909800
H	4.64882000	4.48037800	-1.94219300
H	2.20049400	0.34543400	-0.48497900
O	8.08876700	-0.54229200	-1.70421500
C	9.26158000	-1.25845400	-1.72857900
C	10.45775200	-0.64521000	-2.12008300
C	9.26760300	-2.62102400	-1.42297300
C	11.63221600	-1.39025700	-2.19377500
H	10.45465300	0.41060400	-2.37223900
C	10.45410200	-3.35487700	-1.50178700
H	8.33957500	-3.10365400	-1.13275800
C	11.66499300	-2.76203000	-1.88580300
H	12.54248000	-0.88412400	-2.50198900
H	10.41650300	-4.41031500	-1.25752300
C	12.98918600	-3.54244000	-1.98297700
C	13.52732400	-3.46950300	-3.43181900
H	13.70920000	-2.43767400	-3.74683200
H	14.47417600	-4.01498100	-3.51460800
H	12.81643100	-3.91457800	-4.13588900
C	14.02803800	-2.91736200	-1.02167000
H	14.22475600	-1.86827600	-1.26187100
H	13.67902600	-2.96408100	0.01526300
H	14.97930800	-3.45777500	-1.08474900
C	12.82111100	-5.02691100	-1.60797800
H	12.11842400	-5.53854900	-2.27378800
H	13.78562500	-5.53823200	-1.68997000
H	12.46840300	-5.14984700	-0.57871700
H	2.16753200	4.41706800	-1.92108000
C	-0.61142400	2.38643100	-1.20057500
C	0.60605700	2.38236400	-1.20601900

- SubPc **2**

Coordinates (Angstroms)

Atom	X	Y	Z
N	6.63430600	2.70755300	-0.11201800
C	7.88554000	2.51214000	-0.55402900
C	5.94672300	1.62938300	0.30426100
N	6.53034900	0.39851400	0.45124500
N	8.52747900	1.30643100	-0.44122000
B	8.01442700	0.19944500	0.44203600
C	5.64510100	-0.63887100	0.34763500
N	6.01918100	-1.87345600	-0.02701600
C	7.27597300	-2.03154700	-0.47158300
N	8.21305600	-1.03442400	-0.39911400
C	9.50367900	1.12403400	-1.38312200
N	9.90436000	-0.09419900	-1.78004900
C	9.19820900	-1.14941800	-1.34240700
C	8.68846700	3.29777100	-1.48431700
C	9.70042000	2.42903700	-2.00327100
C	4.51495400	1.39090100	0.36552000
C	4.32231000	-0.02669700	0.39337700
C	9.03979600	-2.48020100	-1.91618000
C	7.83688900	-3.03231900	-1.37123900
C	8.56573300	4.60657700	-1.95670100
C	9.46922300	5.04601700	-2.92157400
C	10.46484700	4.19139200	-3.43191300
C	10.58224000	2.87553900	-2.99029200
C	3.40625300	2.24405700	0.31102600
C	2.13627800	1.68920600	0.31437200
C	1.92445300	0.28242600	0.34487800
C	3.04073300	-0.57098900	0.36890200
C	9.76665400	-3.17771000	-2.88403200
C	9.30294400	-4.42977400	-3.28065500
C	8.11939300	-4.97269100	-2.74500400
C	7.36983000	-4.27751700	-1.79902300
H	9.40350400	6.06441700	-3.29143800
H	7.78924500	5.26170500	-1.57598100
H	11.14962300	4.56550100	-4.18647200
H	11.33988900	2.21324600	-3.39574300
H	10.67031800	-2.75386700	-3.30924500
H	9.86016300	-4.99715200	-4.01945700
H	7.78445400	-5.94931200	-3.08010700
H	6.44984100	-4.69056000	-1.39888000
H	2.90352600	-1.64795800	0.37387100
H	1.28113900	2.35542800	0.28638700
H	3.53919100	3.32014600	0.27227500
O	8.52863300	0.15379000	1.78824300
C	9.86587300	-0.02367400	2.05027800
C	10.38135000	-1.30892500	2.25679800
C	10.71835000	1.07544200	2.17464300
C	11.72688800	-1.47886500	2.57381200
H	9.71890700	-2.16497300	2.17471900
C	12.06619200	0.88855700	2.49246300
H	10.32003400	2.07479800	2.02990000
C	12.60645900	-0.38856000	2.69802800
H	12.09380800	-2.48935000	2.72890300
H	12.69452800	1.76747200	2.58032900
C	14.08663100	-0.62822400	3.04969700
C	14.18367300	-1.34395300	4.41801200

H	13.66861800	-2.30912400	4.40957000
H	15.23212700	-1.52790100	4.67852700
H	13.73893200	-0.73347300	5.21091600
C	14.73804500	-1.51618200	1.96283700
H	14.24026700	-2.48674000	1.87825100
H	14.69363100	-1.03013400	0.98254200
H	15.79112600	-1.70156200	2.20282100
C	14.88602200	0.68550500	3.13795100
H	14.49406000	1.34985800	3.91504200
H	15.92899200	0.46518500	3.38723700
H	14.88122500	1.23011100	2.18806300
N	-6.63427400	-2.70745400	-0.11256900
C	-7.88551000	-2.51194200	-0.55453000
C	-5.94669200	-1.62937900	0.30395900
N	-6.53031800	-0.39854400	0.45125100
N	-8.52744200	-1.30625600	-0.44145100
B	-8.01439800	-0.19947600	0.44206900
C	-5.64506700	0.63886200	0.34784800
N	-6.01914600	1.87353200	-0.02652400
C	-7.27593800	2.03172700	-0.47104800
N	-8.21302400	1.03458600	-0.39879900
C	-9.50365200	-1.12364900	-1.38329600
N	-9.90433600	0.09467700	-1.77994400
C	-9.19817800	1.14979300	-1.34206600
C	-8.68844700	-3.29736400	-1.48498600
C	-9.70040200	-2.42851200	-2.00373600
C	-4.51492200	-1.39091300	0.36523500
C	-4.32227800	0.02667800	0.39341400
C	-9.03976700	2.48070700	-1.91553700
C	-7.83685800	3.03270100	-1.37047500
C	-8.56571900	-4.60606400	-1.95766300
C	-9.46921900	-5.04528700	-2.92262600
C	-10.46484600	-4.19054700	-3.43276500
C	-10.58223200	-2.87479200	-2.99084800
C	-3.40622400	-2.24405600	0.31048300
C	-2.13624900	-1.68920600	0.31390700
C	-1.92442200	-0.28243300	0.34475900
C	-3.04070000	0.57097700	0.36901700
C	-9.76662600	3.17843600	-2.88323100
C	-9.30291500	4.43058900	-3.27957100
C	-8.11936100	4.97338300	-2.74380000
C	-7.36979700	4.27799500	-1.79797800
H	-9.40350500	-6.06360400	-3.29271800
H	-7.78922800	-5.26127700	-1.57709600
H	-11.14962900	-4.56448600	-4.18740100
H	-11.33988300	-2.21240700	-3.39614500
H	-10.67029000	2.75469100	-3.30853800
H	-9.86013500	4.99813500	-4.01824300
H	-7.78442200	5.95008000	-3.07868200
H	-6.44980700	4.69094400	-1.39774400
H	-2.90349500	1.64794400	0.37423200
H	-1.28111100	-2.35542100	0.28568200
H	-3.53916400	-3.32013500	0.27146300
O	-8.52863300	-0.15419600	1.78827900
C	-9.86589300	0.02317400	2.05029700
C	-10.38160600	1.30839700	2.25639300

C	-10.71815700	-1.07606300	2.17506300
C	-11.72717300	1.47818400	2.57337900
H	-9.71934000	2.16455400	2.17402500
C	-12.06602200	-0.88933300	2.49286400
H	-10.31964100	-2.07538500	2.03063300
C	-12.60653000	0.38775500	2.69799400
H	-12.09428000	2.48865200	2.72813900
H	-12.69418800	-1.76833700	2.58105400
C	-14.08673500	0.62725000	3.04963400
C	-14.18386700	1.34344400	4.41769900
H	-13.66900400	2.30871500	4.40889400
H	-15.23234700	1.52727500	4.67819100
H	-13.73897200	0.73333400	5.21080100
C	-14.73837300	1.51468800	1.96248100
H	-14.24079800	2.48531800	1.87753000
H	-14.69389600	1.02830100	0.98235800
H	-15.79148300	1.69993800	2.20244000
C	-14.88585400	-0.68661100	3.13838600
H	-14.49370900	-1.35061900	3.91567800
H	-15.92885400	-0.46641700	3.38766100
H	-14.88100500	-1.23153900	2.18868300
C	-0.59292600	0.32370200	0.34589300
H	-0.59979100	1.41129700	0.35027000
C	0.59295700	-0.32371200	0.34588100
H	0.59981900	-1.41130800	0.35012000

- SubPc **3**

Atom	Coordinates (Angstroms) X	Y	Z
N	-6.67562100	2.45348300	-0.57807100
C	-7.90368100	2.60592300	-0.05974400
C	-6.18734600	1.20377600	-0.66076800
N	-6.95233400	0.09744200	-0.40268900
N	-8.72074500	1.54321300	0.22443800
B	-8.44478200	0.15397900	-0.28805700
C	-6.22253600	-0.99062100	-0.00994600
N	-6.74122800	-1.97750100	0.73834200
C	-7.96917200	-1.78853700	1.24781600
N	-8.75362200	-0.72068100	0.89895000
C	-9.63639200	1.81332900	1.20575900
N	-10.17697800	0.85263400	1.97161400
C	-9.66825700	-0.38537600	1.86071900
C	-8.50792300	3.75411900	0.60608400
C	-9.59181800	3.25806400	1.39805800
C	-4.81374300	0.72871100	-0.68842300
C	-4.83212100	-0.64510700	-0.28478200
C	-9.66056200	-1.48938800	2.81244200
C	-8.59728200	-2.36767100	2.42888200
C	-8.15885700	5.10605000	0.64728200
C	-8.90994100	5.95581000	1.45599000
C	-9.97634000	5.46783200	2.23499800

C	-10.31875700	4.11768800	2.22502400
C	-3.59404900	1.36732600	-0.92853900
C	-3.59404900	1.36732600	-0.92853900
C	-2.42304900	0.63363600	-0.80090700
C	-2.42516400	-0.72930000	-0.40085300
C	-3.64935300	-1.36010200	-0.12184600
C	-10.40293000	-1.73607400	3.96992600
C	-10.09665600	-2.86902100	4.71987300
C	-9.05052700	-3.73280300	4.34280100
C	-8.28437200	-3.48554600	3.20629200
H	-8.66748000	7.01326600	1.49117200
H	-7.32734400	5.48090100	0.05978000
H	-10.53774200	6.15742300	2.85752000
H	-11.13067000	3.74054300	2.83789400
H	-11.19903100	-1.06298300	4.27034300
H	-10.67065300	-3.08968100	5.61433900
H	-8.83612400	-4.60442300	4.95318800
H	-7.46854700	-4.14352000	2.92593900
H	-3.67679300	-2.38485200	0.23120000
H	-1.47670100	1.11432600	-1.02762600
H	-3.56276400	2.41085000	-1.22322400
O	-9.06294100	-0.20921500	-1.53873800
C	-10.42791400	-0.23562800	-1.69538100
C	-11.14620500	-1.41285400	-1.45333900
C	-11.11115700	0.89151400	-2.15665200
C	-12.52228000	-1.44688600	-1.66577900
H	-10.61686500	-2.29551400	-1.10824600
C	-12.49195200	0.84142600	-2.36530300
H	-10.55599000	1.80221800	-2.35865000
C	-13.23336300	-0.32375000	-2.12474500
H	-13.04901800	-2.37622500	-1.46931200
H	-12.98486100	1.73731200	-2.72511700
C	-14.75502800	-0.41235900	-2.34545500
C	-15.06216300	-1.50641500	-3.39558500
H	-14.69974100	-2.48855300	-3.07771000
H	-16.14300500	-1.58648400	-3.55776100
H	-14.59140400	-1.27001000	-4.35564200
C	-15.44946100	-0.77716700	-1.01174100
H	-15.09910800	-1.73672100	-0.61999900
H	-15.25828400	-0.01414800	-0.24985100
H	-16.53352800	-0.85115300	-1.15413100
C	-15.35056500	0.91607100	-2.84855800
H	-14.92213000	1.21653200	-3.81034600
H	-16.43065500	0.80514300	-2.98843200
H	-15.19179500	1.73042600	-2.13405600
N	5.97980300	0.83816500	-0.89796400
C	7.17589500	1.22415200	-1.36992900
C	5.94639200	-0.24801100	-0.10895600
N	7.07998800	-0.85555900	0.35627000
N	8.35057600	0.65892800	-0.94752800
B	8.43117500	-0.21448400	0.27713500
C	6.90493100	-2.18024300	0.65692100
N	7.92067900	-3.06103700	0.65062300
C	9.10046900	-2.64311000	0.16771400
N	9.34264900	-1.33329400	-0.15477400
C	9.35730800	0.75267600	-1.87018100

N	10.38700300	-0.10886900	-1.90403900
C	10.32106300	-1.18181200	-1.10005800
C	7.51437600	1.98458900	-2.56675500
C	8.87957600	1.68948100	-2.87973400
C	4.86520400	-1.19055100	0.15429600
C	5.46801700	-2.39816900	0.63022200
C	10.96075300	-2.48698800	-1.21639800
C	10.19738600	-3.40132800	-0.42303100
C	6.75242600	2.79746100	-3.40963400
C	7.36520800	3.33046700	-4.54114600
C	8.70832300	3.04060500	-4.84867700
C	9.47255300	2.21008700	-4.03255500
C	3.49277700	-1.11422800	-0.06752400
C	2.68944100	-2.22997000	0.21751200
C	4.67231700	-3.51969200	0.88482000
C	12.03925500	-2.93857600	-1.98067400
C	12.36366500	-4.29212200	-1.92669600
C	11.61263700	-5.19169800	-1.14640800
C	10.51816000	-4.76069800	-0.40028600
H	6.79746300	3.97798000	-5.20192900
H	5.71282200	3.00919100	-3.18274900
H	9.15286700	3.46975800	-5.74112000
H	10.50261900	1.97510500	-4.27916100
H	12.60913700	-2.24922500	-2.59478400
H	13.20781500	-4.66308100	-2.49943100
H	11.89068400	-6.24084000	-1.13095600
H	9.93098300	-5.45801900	0.18798500
H	5.11510200	-4.44429600	1.23978100
H	3.05929500	-0.20419300	-0.46928400
O	8.75401800	0.44087900	1.51974500
C	9.93703200	1.11587000	1.70196300
C	11.05572300	0.45667200	2.22576000
C	10.02415900	2.48204800	1.42604100
C	12.23510600	1.16035700	2.45791100
H	10.98815700	-0.60212300	2.45544100
C	11.21431700	3.17415500	1.66475000
H	9.15490700	3.00010200	1.03313100
C	12.34910500	2.53487200	2.18319100
H	13.08374500	0.61893700	2.86593400
H	11.24051500	4.23394600	1.43848500
C	13.67504700	3.26873800	2.45777500
C	14.02836600	3.15394000	3.95977300
H	14.13815400	2.11160200	4.27348100
H	14.97421800	3.66627300	4.16905800
H	13.25031800	3.61063400	4.58045400
C	14.80425900	2.62539900	1.61815100
H	14.93621900	1.56613000	1.85789800
H	14.58760000	2.70229600	0.54749200
H	15.75718900	3.13171900	1.80924100
C	13.60080200	4.76403800	2.09570300
H	12.83772400	5.28770900	2.68080100
H	14.56335400	5.24134900	2.30583700
H	13.38155700	4.91584100	1.03373900
C	-1.17412500	-1.48025200	-0.27318100
C	-0.98384100	-2.84556000	-0.32993100
C	0.36544200	-3.24320500	-0.16284500

C	1.23825000	-2.19072500	0.02306800
S	0.35813500	-0.67361000	0.00565000
H	-1.78867700	-3.54520200	-0.52242700
H	0.68819100	-4.27619800	-0.21579900
C	3.30166900	-3.42025900	0.69708300
H	2.67519900	-4.27204500	0.93762100

- SubPc **4**

	Coordinates (Angstroms)		
Atom	X	Y	Z
N	-7.22718500	2.69348300	-0.22946300
C	-8.50609700	2.43935000	0.08837100
C	-6.44065500	1.64167600	-0.50236800
N	-6.93175800	0.37027500	-0.63484700
N	-9.06291100	1.19437000	-0.01429500
B	-8.39389800	0.07604700	-0.77703500
C	-6.00358100	-0.59939800	-0.37566300
N	-6.33971300	-1.83176200	0.02927300
C	-7.62755400	-2.04466300	0.34640200
N	-8.60926600	-1.12039700	0.11900500
C	-10.12710900	0.99584800	0.82327600
N	-10.49753600	-0.21918600	1.25108900
C	-9.68763200	-1.24538700	0.95288700
C	-9.45234900	3.21931600	0.87180700
C	-10.46689600	2.31614300	1.33101600
C	-4.99583600	1.49564600	-0.40409800
C	-4.71853100	0.09361500	-0.32577000
C	-9.51543600	-2.53014500	1.61255100
C	-8.22564700	-3.02978100	1.23382700
C	-9.46628800	4.54975600	1.28063100
C	-10.48586300	4.98913200	2.11765200
C	-11.47858100	4.10516500	2.56689300
C	-11.46759800	2.76780200	2.18632200
C	-3.96076900	2.42782800	-0.29369200
C	-2.66348400	1.96078600	-0.13701500
C	-2.37699600	0.57008800	-0.05982900
C	-3.42122600	-0.37059700	-0.14082600
C	-10.28896400	-3.22627000	2.53696000
C	-9.80220000	-4.41661000	3.06546300
C	-8.54016900	-4.90553400	2.69496600
C	-7.74478100	-4.21174100	1.79018600
H	-3.21151300	-1.43140600	-0.06474400
H	-1.84061100	2.66343000	-0.06632600
H	-4.16602300	3.49195900	-0.33577500
O	-8.75360900	-0.07346900	-2.15686700
C	-10.04534900	-0.34188900	-2.55203600
C	-10.47245300	-1.66412200	-2.71787500
C	-10.92534200	0.69960200	-2.85038800
C	-11.76459400	-1.92701200	-3.16653200
H	-9.78445100	-2.47586200	-2.50327100
C	-12.21856300	0.41908400	-3.29929500
H	-10.59127000	1.72649300	-2.73975800

C	-12.67313200	-0.89637900	-3.46648900
H	-12.06516000	-2.96379100	-3.28595100
H	-12.87224400	1.25467200	-3.52125400
C	-14.09190700	-1.23841600	-3.95828400
C	-14.00171400	-2.05122100	-5.27173700
H	-13.44864800	-2.98524500	-5.13517800
H	-15.00497300	-2.30753600	-5.63025000
H	-13.49835000	-1.47465600	-6.05489700
C	-14.81974100	-2.08264300	-2.88515200
H	-14.29091600	-3.01703000	-2.67488000
H	-14.90816500	-1.52838700	-1.94488400
H	-15.82948900	-2.34027900	-3.22394200
C	-14.93497200	0.02163000	-4.23047500
H	-14.48921200	0.65059100	-5.00802400
H	-15.93225400	-0.27095600	-4.57415800
H	-15.06167100	0.62992700	-3.32891300
C	-1.03280600	0.13467200	0.10348100
N	5.40271400	1.78732700	0.37438700
C	6.68354000	2.02255500	0.05837400
C	5.07778200	0.53901000	0.75382600
N	6.01614800	-0.42721800	0.98600600
N	7.67617100	1.09833400	0.26178400
B	7.47677300	-0.12047600	1.12488200
C	5.53880200	-1.70138100	0.83026900
N	6.34002300	-2.73983000	0.52924700
C	7.61227500	-2.46751100	0.21210100
N	8.15470200	-1.21384500	0.34080400
C	8.74012300	1.25413300	-0.58530400
N	9.55980000	0.24121600	-0.90930500
C	9.20375100	-0.99008400	-0.50905200
C	7.26691700	3.03402600	-0.81666100
C	8.54827600	2.55124200	-1.21702600
C	3.80392100	-0.16545100	0.68462200
C	4.09556700	-1.56799300	0.73252200
C	9.55029300	-2.29515500	-1.05242200
C	8.56137300	-3.21967500	-0.60259400
C	6.75240700	4.22218800	-1.33213600
C	7.52499100	4.96261500	-2.23467400
C	8.81576500	4.48696600	-2.62478600
C	9.30816700	3.27879400	-2.13361700
C	2.49989300	0.28743600	0.50726300
C	1.46677000	-0.66064700	0.40355100
C	3.06690000	-2.50733300	0.60146200
C	10.54742200	-2.71887300	-1.93166700
C	10.58222400	-4.05274000	-2.33519500
C	9.58393000	-4.97604500	-1.89331900
C	8.56883800	-4.54593000	-1.03050100
H	5.76611000	4.54923400	-1.02983900
H	10.27821800	2.92098400	-2.46033600
H	11.29820200	-2.02676900	-2.29651400
H	7.79037000	-5.21762200	-0.69273200
H	3.28224000	-3.57025000	0.62170200
H	2.27874100	1.34742800	0.45366400
O	7.83806800	0.00130800	2.51347800
C	9.12480600	0.27907100	2.91009000
C	10.01858300	-0.76117000	3.19097300

C	9.54072000	1.59873400	3.09773400
C	11.30391300	-0.47335100	3.64376200
H	9.69439000	-1.78888000	3.06024500
C	10.83363000	1.87118200	3.55251300
H	8.84606600	2.40795900	2.89529300
C	11.74822800	0.84711400	3.83518600
H	11.97190800	-1.30363200	3.85367900
H	11.11763300	2.90868900	3.68637300
C	13.17917600	1.11371300	4.33840700
C	13.37285600	0.44909000	5.72218900
H	13.21705900	-0.63298600	5.67923100
H	14.38997300	0.62487400	6.09022300
H	12.67076700	0.86011600	6.45520300
C	14.19753800	0.51534300	3.33894000
H	14.06684100	-0.56465800	3.22269100
H	14.09129200	0.97430900	2.35038600
H	15.22158000	0.69144000	3.68705300
C	13.47644000	2.61814200	4.48304600
H	12.80712000	3.09853700	5.20422500
H	14.50170800	2.75747200	4.84053200
H	13.38514800	3.14529100	3.52769800
F	-6.54244900	-4.70005800	1.46781400
F	-8.11165800	-6.05543000	3.22474500
F	-10.53565300	-5.11628200	3.93647100
F	-11.49084800	-2.78376500	2.92021800
F	-12.42269600	1.95045200	2.64099200
F	-12.44266100	4.56930400	3.36764200
F	-10.53530900	6.26740700	2.50493400
F	-8.53030800	5.41651500	0.87980600
S	11.87428000	-4.55967700	-3.47587300
S	9.68831900	-6.64946400	-2.49131200
S	9.81881200	5.39147700	-3.80932500
S	6.96095800	6.49344500	-2.94723500
C	10.62628600	6.63766100	-2.72636900
H	9.88231000	7.28190600	-2.25593100
H	11.24098400	6.14615200	-1.97088500
H	11.26315000	7.23767100	-3.37978900
C	5.35311200	6.76908500	-2.12808900
H	4.99499100	7.72074600	-2.52504400
H	4.63317300	5.98797100	-2.37985600
H	5.46330400	6.85409900	-1.04525700
C	13.10366500	-5.30027200	-2.32780600
H	12.67685700	-6.15584800	-1.80271500
H	13.93466600	-5.63640200	-2.95152200
H	13.46108500	-4.55333400	-1.61748500
C	8.31750700	-7.48960100	-1.62701300
H	8.43866100	-7.44491300	-0.54295600
H	7.34714900	-7.08515200	-1.92124200
H	8.37861100	-8.53077200	-1.94907500
C	1.76613000	-2.05127000	0.45257300
H	0.95004200	-2.76007000	0.36527900
C	0.11939700	-0.23248900	0.24337800
